# ToTem: a tool for variant calling pipeline optimization

**DOI:** 10.1186/s12859-018-2227-x

**Published:** 2018-06-26

**Authors:** Nikola Tom, Ondrej Tom, Jitka Malcikova, Sarka Pavlova, Blanka Kubesova, Tobias Rausch, Miroslav Kolarik, Vladimir Benes, Vojtech Bystry, Sarka Pospisilova

**Affiliations:** 1 Center of Molecular Medicine, Central European Institute of Technology, Masaryk University, Brno, Czech Republic; 2 Department of Internal Medicine - Hematology and Oncology, Medical Faculty, Masaryk University and University Hospital Brno, Brno, Czech Republic; 3 Department of Computer Science, Faculty of Science, Palacky University, Olomouc, Czech Republic; 4 Genomics Core Facility, European Molecular Biology Laboratory, Heidelberg, Germany

**Keywords:** Variant calling, Benchmarking, Next generation sequencing, Parameter optimization

## Abstract

**Background:**

High-throughput bioinformatics analyses of next generation sequencing (NGS) data often require challenging pipeline optimization. The key problem is choosing appropriate tools and selecting the best parameters for optimal precision and recall.

**Results:**

Here we introduce ToTem, a tool for automated pipeline optimization. ToTem is a stand-alone web application with a comprehensive graphical user interface (GUI). ToTem is written in Java and PHP with an underlying connection to a MySQL database. Its primary role is to automatically generate, execute and benchmark different variant calling pipeline settings. Our tool allows an analysis to be started from any level of the process and with the possibility of plugging almost any tool or code. To prevent an over-fitting of pipeline parameters, ToTem ensures the reproducibility of these by using cross validation techniques that penalize the final precision, recall and F-measure. The results are interpreted as interactive graphs and tables allowing an optimal pipeline to be selected, based on the user’s priorities. Using ToTem, we were able to optimize somatic variant calling from ultra-deep targeted gene sequencing (TGS) data and germline variant detection in whole genome sequencing (WGS) data.

**Conclusions:**

ToTem is a tool for automated pipeline optimization which is freely available as a web application at https://totem.software.

**Electronic supplementary material:**

The online version of this article (10.1186/s12859-018-2227-x) contains supplementary material, which is available to authorized users.

## Background

NGS is becoming the method of choice for an ever-growing number of applications in both research and clinics [1]. However, obtaining unbiased and accurate NGS analysis results usually requires a complex multi-step processing pipeline, specifically tailored to the data and experimental design. In the case of variant detection from DNA sequencing data, the analytical pipeline includes pre-processing, read alignment and variant calling. Multiple tools are available for each of these steps, each using its own set of modifiable parameters, creating a vast amount of possible distinct pipelines which vary greatly in the resulting called variants [2]. Selecting an adequate pipeline is a daunting task for a non-professional, and even an experienced bioinformatician needs to test many configurations in order to optimize the analysis.

To resolve this complexity, modern variant calling approaches utilize machine learning algorithms to automatically tune the analysis. However, the machine learning approaches often require a large number of samples. According to GATK Best practices, Variant Quality Score Recalibration (VQSR) [3, 4], which is widely used for variant filtration, requires > 30 whole exomes and at least basic parameter optimization. Variant calling on small scale data, e.g. gene panels which are very often used in diagnostics, still needs to be done with fixed thresholds, reiterating the aforementioned problem of an optimal workflow configuration.

The evaluation of current variant calling pipelines [5, 6] and the development of benchmarking toolkits [7, 8] have helped to resolve this task, but to the best of our knowledge, there is no tool enabling automated pipeline parameter configuration using a ground truth data set.

In this paper, we present ToTem, a method for pipeline optimization which can automatically configure and benchmark individual tools or entire workflows, based on a set of validated ground truth variants. In this way, ToTem helps to choose the optimal pipeline for specific needs. The applicability of ToTem was demonstrated using two common NGS variant calling tasks: (1) Optimal somatic variant calling using ultra-deep TGS data and (2) optimal germline variant calling using WGS data. In both scenarios, we were able to significantly improve the variant calling performance in comparison to the tools’ default settings.

## Implementation

ToTem is a stand-alone web application with a comprehensive GUI which allows ToTem to be used even by non-bioinformaticians, and for advanced users it features a convenient pipeline editor which takes care of parallelization and process control. The server backend is implemented in Java and PHP with an underlying connection to the MySQL database. All communication with the server is encrypted.

ToTem is primarily intended for testing variant calling pipelines with the ability to start an analysis from any level of the process. This allows testing either whole pipelines starting from raw sequencing data or focussing only on the final variant filtering phases. The results are visualized as interactive graphs and tables. ToTem also provides several convenient auxiliary tools that facilitate maintenance, backup and input data source handling.

### Pipeline configuration and execution

The core principle of pipeline optimization in ToTem is to automatically test pipeline performance for all the parameter combinations in a user defined range. Pipelines are defined through consecutively linked “processes”, where each process can execute one or more tools, functions or code. ToTem is optimized to test the pipelines represented as linear sequences of commands, but also supports branching at the level of tested processes, e.g. to simultaneously optimize two variant callers in one pipeline. To facilitate pipeline definition, common steps shared by multiple pipelines can be easily copied or moved using drag and drop function.

Processes are constructed from template scripts that use bash script code with special syntax to include placeholders for automatic testing. From ToTem’s pipeline optimization concept’s point of view, the most important placeholder, called “params”, is dedicated to inserting the tested parameters to be optimized. Each parameter can be represented simply by their presence or absence, one value, more values, intervals or even mathematical functions. Parameter ranges can be easily set through GUI without the necessity to scan or modify a code. Therefore, with prepared templates, the scope and focus of the optimization can easily be changed without informatics proficiency. ToTem provides predefined templates for the tools most commonly used in variant-calling pipelines.

When a pipeline framework for testing is prepared, input data can be uploaded to the attached storage via GUI, where they are accessible through several placeholders designed for particular data types. When the analysis is started, ToTem creates all possible pipelines within the preset parameter ranges and executes them on the attached computational server. All the processes for combined settings are executed in parallel, limited by a defined maximal number of threads. The parallelization, resource control and asynchronous communication with the application server are managed by ToTem’s backend. The results are imported into ToTem’s internal database for final evaluation and benchmarking. The analysis time depends on the available computational power, the level of parallelization, performance of the particular tool, the number of tested configurations and the size and nature of the input data. For technical details and practical examples, see Additional file 1 and watch step-by-step tutorial on totem.software web pages.

### Pipeline benchmarking

The benchmarking of each pipeline is done using ground truth data and is based on an evaluation of true positives, false positives, false negative rates and performance quality metrics derived from them. Ground truth data generally consists of raw sequencing data or alignments and an associated set of validated variants [9, 10].

ToTem provides two benchmarking approaches, with each focusing on different applications and having different advantages:

- The first approach is using ToTem’s filtering tool to filter (stratified) performance reports generated by external benchmarking tools, which are incorporated as a final part of tested analytical pipelines. This allows an evaluation of many parameter combinations and simple setting selection that produce the best results considering e.g. quality metrics, variant type and region of interest (variables depend on the report). This approach is particularly useful for optimizing the pipeline for WGS or whole exome sequencing (WES) and also TGS.
- Little Profet (LP) is ToTem’s genuine benchmarking method, which compares variant calls generated by tested pipelines to the gold standard variant call set. LP calculates standard quality metrics (precision, recall and F-measure) and most importantly – the reproducibility of each quality metric, which is the main advantage over the standard Genome in a Bottle (GIAB) approach. ToTem thus allows the best pipelines to be selected considering the selected quality metrics and its consistency over multiple data subsets. The LP approach is designed primarily for TGS data harbouring a limited number of sequence variants and suffering from high a risk of pipeline over-fitting.

#### ToTem’s filtering tool for Genome in a Bottle benchmarking approach

The GIAB benchmarking approach, which combines RTG Tools [11, 12] and hap.py [13], is best suited to variant calling pipelines designed for the data which might harbour complex variants and require variant and region stratification, e. g. WGS data. RTG Tools use complex matching algorithms and standardized counting applied for variant normalization and comparison to the ground truth. Hap.py is applied for variant and region annotation/stratification [14]. These tools serve as reference implementations of the benchmarking standards agreed upon by the ga4gh data working group [15]. Regarding ToTem’s pipeline optimization concept, RTG Tools and hap.py are used to be a final part of the pipeline providing, as a result, a regionally stratified performance (precision, recall, F-measure, etc.) report for several variant types.

The reports from all pipeline configurations are imported into the internal database and processed by ToTem’s filtering tool, allowing easy selection of an optimal pipeline based on the user’s needs and priorities. This could be extremely useful while ranking the pipelines for a specific variant type, e.g. single nucleotide variant (SNV) versus insertion or deletion (InDel), variant calling filters and/or specific regions of the genome such as low-mappability regions, low-complexity regions, AT-rich regions, homopolymers, etc. described as significantly influencing variant calling performance [16–18]. The complete list of filtered results describing the performance qualities for the selected variant type and region for all the pipelines can be exported into a csv table for deeper evaluation.

**Fig. 1:**
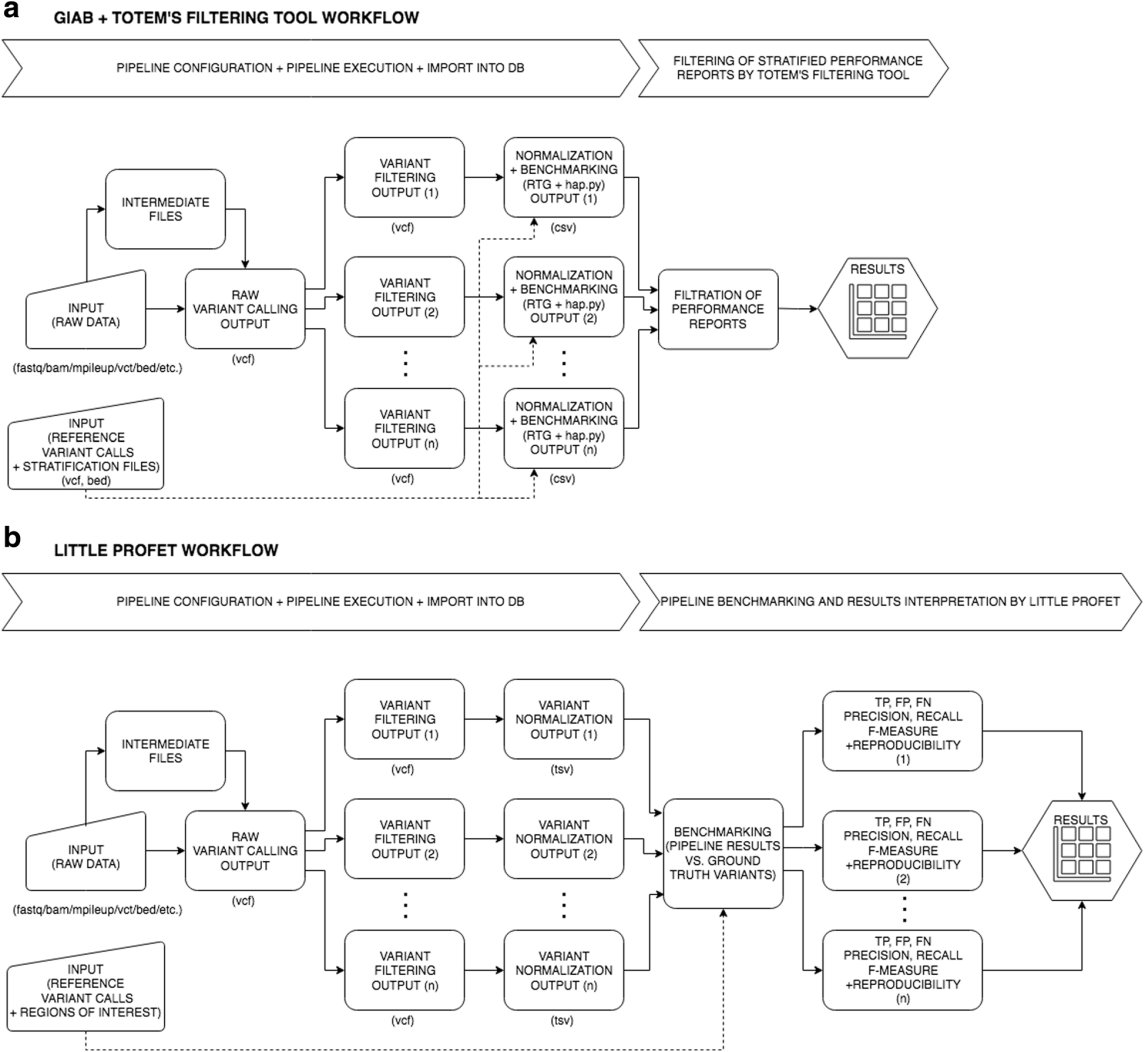
**a** Once the pipeline is set up for the optimization, all the configurations are run in parallel using raw input data. In this particular example, the emphasis is placed on optimizing the variant calling filters, however, the pipeline design depends on the user’s needs. In the case of the GIAB approach, the benchmarking step is part of the pipeline done by RTG Tools and hap.py. The pipeline results in the form of the stratified performance reports (csv) provided by hap.py are imported into ToTem’s internal database and filtered using ToTem’s filtering tool. This allows the best performing pipeline to be selected based on the chosen quality metrics, variant type and genomic region. **b** Similar to the previous diagram, the optimization is focused on tuning the variant filtering. Contrary to the previous case, Little Profet requires the pipeline results to be represented as tables of normalized variants with mandatory headers (CHROM, POS, REF, ALT). Such data are imported into ToTem’s internal database for pipeline benchmarking by the Little Profet method. Benchmarking is done by comparing the results of each pipeline to the ground truth reference variant calls in the given regions of interest and by estimating TP, FP, FN; and quality metrics derived from them - precision, recall and F-measure. To prevent overfitting of the pipelines, Little Profet also calculates the reproducibility of each quality metric over different data subsets. The results are provided in the form of interactive graphs and tables

ToTem’s filtering tool utility is not only restricted to the GIAB approach but can also be applied to other table formats describing pipeline performance. The specific format, e.g. column names, column separator, needs to be set through the ToTem GUI before importing pipeline results into the database. ToTem’s fitering workflow is described in Fig. 1, part A. For technical details and practical examples, see Additional file 1 and watch step-by-step tutorial on totem.software web pages.

#### Benchmarking by Little Profet

The weakness of pipeline optimization using a ground truth data set is that it may lead to an over-fit of the parameters causing inaccuracies when analyzing a different dataset. This negative effect is even more pronounced when using small scale data like TGS, usually harboring a relatively small number of ground truth variants.

To address this task, ToTem proposes its genuine benchmarking algorithm, LP, which prevents over-fitting and ensures the pipeline reproducibility. LP therefore represents an alternative to the GIAB approach with the added value of taking additional measures to guarantee robust results.

The LP benchmarking is based on the comparison of the normalized variants detected by each pipeline to the ground truth reference variants in the regions of interest and the inferred precision, recall and F-measure.

The over-fitting correction utilizes cross validation approaches that penalize the precision, recall and F-measure scores based on the result variation over different data subsets. The assumption is that the pipelines showing the least variability of results among data subsets will also prove to be more robust when applied to unknown data.

The reproducibility is calculated from all the samples (> 3) going into the analysis, while a repeated (number of repeats = ½ of samples) random sub-sampling (number of samples in one sampling group = ½ of samples) validation is performed to estimate the sub-sampling standard deviation (SMSD) of the validation results for individual performance quality metrics (precision, recall and F-measure). The reproducibility may also be inferred from the min/max values for a given performance quality measure calculated for each sub-sampling group. If multiple distinct data sets are provided (at least 2), standard deviation between the selected data set results (DSD) can be used to assess reproducibility as well.

Additionally, to improve the precision and consistency of variant detection [19], the intersection of the results from each pair of 10 best performing pipelines (5 pipelines with higher precision, 5 with higher recall) is done by default. The detailed information about pipeline performance including over-fitting correction can be exported to excel file for further evaluations. Little Profet workflow is described in Fig. 1, part B. To better understand LP method, pseudo code is provided in Additional file 2. For other technical details and practical examples, see Additional file 1 and watch step-by-step tutorial on totem.software web pages.

## Results

To showcase the advantages and versatility of ToTem, we performed the optimization test of variant calling pipelines for two very diverse experimental settings:

- somatic variant calling on ultra-deep TGS data
- germline variant calling on WGS data.

In the first setting, we used ultra-deep targeted gene sequencing data from the *TP53* gene (exons 2–11) from 220 patient samples divided into 3 data sets based on differences in diagnosis, verification status and mutation load. A combination of three datasets was used in the context of the Little Profet over-fitting control capability, ensuring the robustness of the particular pipeline settings applied to a slightly different type of data. One thousand twelve manually curated variants with a variant allele frequency (VAF) ranging from 0.1 to 100% were used as ground truth variant calls for pipeline benchmarking [20, 21].

All DNA samples were sequenced with ultra-high coverage (min. coverage depth > 5000×, average depth of coverage approx. 35 000×) using Nextera XT DNA Sample Preparation Kit and MiSeq Reagent Kit v2 (300 cycles) (Illumina, San Diego, CA, USA) on a MiSeq instrument, as described previously [20]. Reads’ quality trimming, merging and mapping onto the reference genome (GRCh37) as well as variant calling, was done using CLC Genomic Workbench. The Shearwater algorithm from the R-package DeepSNV, computing a Bayes classifier based on a beta-binomial model for variant calling with multiple samples to precisely estimate model parameters - such as local error rates and dispersion, [22] was used as the second variant calling approach. The minimum variant read count was set to 10. Only variants detected either by both variant calling algorithms or confirmed by a technical or biological replicate were added to the list of candidate ground truth variants. To remove remaining FP, filtering was applied according to VAF present in an in-house database containing all the samples processed in our laboratory. Because an in-house database accumulates false-positive variants specific for the used sequencing platform, sequencer and analysis pipeline, it could be used to identify and remove these FP. All computationally predicted variants were manually checked by expert users and confirmed by biological findings [20, 21]. This approach allowed us to detect variants down to 0.1% VAF.

Only SNV were considered during the analysis. Short InDels were not included in the ground truth set due to their insufficient quantity.

Dataset TGS 1 was represented by 355 SNVs detected in 103 samples from patients diagnosed with chronic lymphocytic leukemia (CLL). The dataset represented variants detected in VAF ranging from 0.1–100%. Variant calling was done by CLC Genomic Workbench and Shearwater algorithm. Only variants confirmed by both algorithms or by a biological/technical replicate were taken into account. The dataset should not contain any false positive variants.

Dataset TGS 2 consisted of 248 SNVs present in 77 patient samples with myeloproliferative neoplasm (MPN). With the exception of known germline polymorphisms, variants representing low burden sub-clones up to 10% VAF prevailed, as fully expanded (> 20%VAF) *TP53* mutations are rare in MPN [21]. Only variants detected by CLC Genomic Workbench, confirmed by technical replicates or by independent sampling were used. The dataset should not contain any false positives variants.

Dataset TGS 3 was represented by 409 SNVs detected in 40 patient samples with CLL with VAF 0.1–100%. Variant calling was done using CLC Genomic Workbench only and false positive variants may rarely occur as some of the low frequency variants were not confirmed by a technical replicate, for more details see Additional file 3.

In the first experiment, three variant callers were optimized: Mutect2 [3, 4], VarDict [23] and VarScan2 [24, 25], using all 3 TGS datasets. Aligned reads generated outside of ToTem with the BWA-MEM algorithm [26] were used as input data for the pipeline optimization, which was focused on tuning the variant callers’ hard filters. As part of the optimized pipeline, variants passing filters were normalized by vcflib [27], imported into the internal database and processed using Little Profet. The pipelines’ performance was sorted by F-measure corrected by SMSD. A detailed description of the pipelines including their configurations can be found in Additional file 3.

The best results were achieved using optimized VarScan2, specifically by intersecting the results generated by two different settings, reaching a precision of 0.8833, recall of 0.8903 and an F-measure of 0.8868. This precision is high considering the tested datasets contained 624 variants with very low VAF (< 1%), which are generally problematic to identify because of sequencing errors. The importance of ToTem is even more pronounced when compared to the median scoring pipeline, which had a precision of 0.5405, a recall of 0.7527 and an F-measure of 0.6292, and compared to the baseline VarScan2 pipeline using its default parameters, which had a precision of 0.9916, recall of 0.2312 and an F-measure of 0.3763. The best-scoring pipeline thus identified 3.84-fold more true positive variants and showed only an 11% lower precision than the VarScan2 pipeline using default parameters.

The input mpileup files were generated using very sensitive settings allowing the optimization of 4 parameters in 54 different combinations including their default values, for details, see Additional file 3. Compared to the default settings, the detection quality of the best scoring pipeline was affected by tuning all 4 parameters. Higher recall was caused by lowering the parameters for *the minimum variant allele frequency* and *p-value*. High precision was maintained by increasing the parameter values for *the minimum base quality* and *the minimum number of variant supporting reads*.

The second best performing variant caller in our test was VarDict. VarDict parameter optimization was, in principle, similar to VarScan2 – raw variant calling was done using very sensitive settings allowing the testing of hard filter parameters.

The optimized settings achieved a precision of 0.8903, recall of 7468 and an F-measure of 0.8123. Compared to the default settings (a precision of 0.9483, recall of 0.3083 and an F-measure of 0.4653), the quality of detection (F-measure) was improved by 42.7%.

In total, 7 parameters were optimized by assessing 192 of their combinations, including the default values, for details, see Additional file 3. Compared to the default settings, the optimized caller had a decreased parameter for *the minimum allele frequency*, which led to its higher recall. This setting was apparently balanced by increasing *the minimum high quality variant depth*, which works towards a higher precision. The parameters for *the maximal distance for proximity filter*, the *minimum mean base quality* and *the maximum mean mismatches* performed best with their default values. The other parameters had no impact on the analysis results in the tested ranges.

Mutect2 variant calling optimization was done without applying the “FilterMutectCalls” function, because testing several of this function’s parameters, including the default settings, led in our case to rapidly decreased recall and thus to decreased overall performance. Some of the parameters from the “FilterMutectCalls” function are also available as a part of the Mutect2 raw variant calling and were the subject of testing. The best optimized settings thus reached a precision of 0.8397, recall of 0.7567 and an F-measure of 0.7960, whereas the default settings offered a precision of 0.4826, recall of 0.7714 and an F-measure of 0.5937, which was the highest recall and F-measure of all the default settings for all the tested variant callers.

The variant calling optimization tested 36 combinations of 4 parameters including their default values. For details, see Additional file 3. The best Mutect2 pipeline was very similar to the default settings with only one parameter value increased (*the minimum base quality required to consider a base for calling*) towards higher precision. The values of the other parameters remained unchanged or had no effect on the results.

**Fig. 2:**
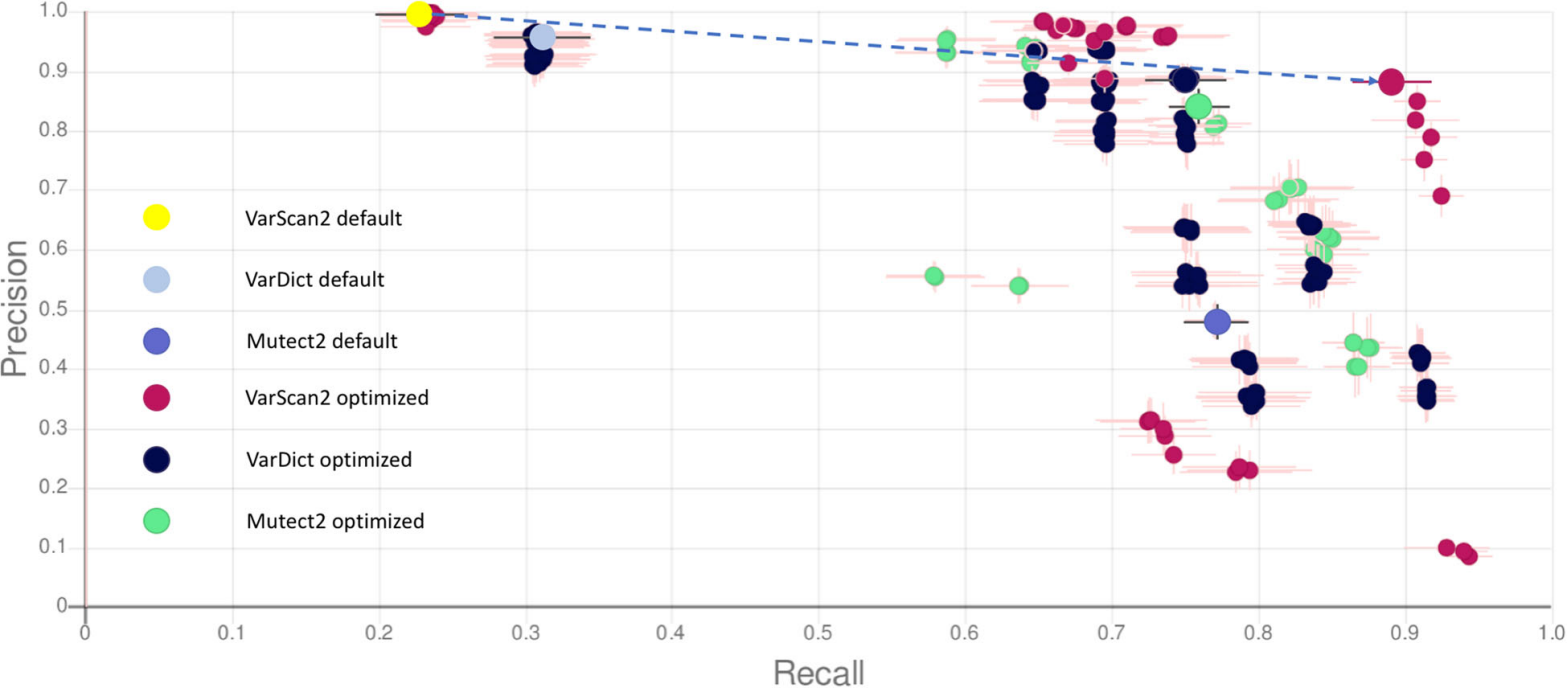
Each dot represents an arithmetic mean of recall (X-axis) and precision (Y-axis) for one pipeline configuration calculated based on repeated random sub-sampling of 3 input datasets (220 samples). The crosshair lines show the standard deviation of the respective results across the sub-sampled sets. Individual variant callers (Mutect2, VarDict and VarScan2) are colour coded with a distinguished default setting for each. The default settings and the best performing configurations for each variant caller are also enlarged. Based on our experiment, the largest variant calling improvement (2.36× higher F-measure compared to default settings, highlighted by an arrow) and also the highest overall recall, precision, precision-recall, and F-measure were registered for VarScan2. In case of VarDict, a significant improvement in variant detection, mainly for recall (2.42×) was observed. The optimization effect on Mutect2 had a great effect on increasing the precision (1.74×). Although the F-measure after optimization did not reach as high values as VarScan2 and VarDict, Mutect2’s default setting provided the best results, mainly in a sense of recall

The graphical interpretation for different pipeline configuration performance for all 3 variant callers and the demonstration of the optimization effect is visualized in Fig. 2; for a detailed performance report exported from LP, see Additional file 4.

In the second experiment, we tested pipeline optimization for germline variant calling using GATK HaplotypeCaller followed by VQSR and VarDict on 2 whole genomes. As reference samples with high-confident variant calls were used NA12878 and HG002 genomes analyzed by GIAB, hosted by the National Institute of Standards and Technology (NIST) which creates reference materials and data for human genome sequencing [10].

As an input for the WGS analysis, BAM files downloaded from the GIAB ftp server were used. Alignments were preprocessed using GATK best practices (removing duplicates, adding read groups, base quality score recalibration) and downsampled to 30× coverage, for details see Additional file 3.

Raw variant calling was done by each variant caller to produce intermediate results representing an input for variant filtering optimization in ToTem, considering both, SNV and InDels. In the case of GATK HaplotypeCaller, the emphasis was placed on tuning the VQSR using machine learning algorithms. In the case of VarDict, hard filters were tuned, for details see Additional file 3.

The filtered variants were compared to the ground truth variant calls by RTG Tools in given high confidence regions. Information about the pipelines’ performance (precision, recall, F-measure, etc.) was stratified into variant sub-types and genomic regions by hap.py. The results in the form of a quality report for each pipeline were imported into ToTem’s internal database and filtered using ToTem’s filtering tool, which allows the best performing pipeline to be selected based on region, variant type and quality metrics.

The best results were achieved by GATK HaplotypeCaller, with a precision of 0.9993, recall of 0.9989 and F-measure of 0.9991 for SNV, and 0.9867, 0.9816 and 0.9842 for InDels, respectively. In comparison to the default settings, a total of 123,716 more TP and 1889 less FP were registered after the optimization by ToTem, where 40 combinations of 2 parameters were tested for both variant types, for details, see Additional file 3. An evident impact on the results’ quality was proven by both of them. Increased values of the parameter for *the truth sensitivity level* influenced the detection of SNP and InDels towards higher recall. The parameter for *the maximal number of Gaussians* only needed to be optimised for InDel detection towards the lower values, otherwise the first VQSR step would not finish successfully for the NA12878 sample.

In the case of VarDict, the best pipeline setting reached a precision of 0.9977, a recall of 0.8597 and F-measure of 0.9236 for SNP; and 0.8859, 0.8697 and 0.8778 for InDels, respectively. Compared to the default settings, the results were improved by identifying 17,985 more TP and 183,850 less FP. In total, 6 parameters were tested in 216 combinations. For details, see Additional file 3.

The improved variant quality detection was affected mainly by the increasing *the minimum allele frequency values*, leading towards higher precision while increasing *the maximum mean mismatches* was responsible for higher recall in SNP detection. InDels calling was also improved by increasing *the minimum mean position of the variants in the read*, which supported higher pipeline precision. The other parameters remained unchanged for the best performing pipeline. The difference between the best pipeline for every tool and the baseline for that tool using default parameters is described in Additional file 5.

The TGS experiment optimizing 3 variant callers was run in parallel by 15 threads (15 parameter combinations running simultaneously) and was completed in approximately 60 h; WGS experiment optimizing 2 variant callers was run using 5 threads and lasted approximately 30 h. The experiments were performed separately on a server with 100 CPU cores and 216 GB RAM memory available, however the server was not used to its full capacity.

## Discussion

ToTem is a web application with an intuitive GUI primarily designed for automated configuration and evaluation of variant calling pipeline performance using validated ground truth material. Once the pipeline is optimized for specific data, project, kit or diagnosis, it can be effortlessly run through ToTem for routine data analysis with no additional need for ground truth material. From this perspective, ToTem represents a unique hybrid between a workflow manager like bcbio [28], SeqMule [19] or Galaxy [29] and a pipeline benchmarking tool like SMaSH [7], with the added value of an automated pipeline generator.

To meet the latest best practices in variant calling benchmarking, ToTem is perfectly suited and fully compatible with the current GIAB approach using RTG Tools and hap.py. This allows comfortable automated parameter optimization, benchmarking and selection of the best pipeline based on variant type, region stratification and preferred performance quality metrics.

The Little Profet benchmarking approach introduces novel estimates of pipeline reproducibility based on a cross validation technique allowing the selection of a robust pipeline that will be less susceptible to over-fitting.

ToTem is also very robust in terms of implementing various tools by its “template approach” allowing the integration and running of any tool or even more importantly, custom or novel code without having to create a special wrapper. These properties enable automatic and significantly less biased testing for new or existing variant calling pipelines than standard procedures, testing only the default or just a few alternative settings [5, 6].

The results are visualized through several interactive graphs and tables enabling users to easily choose the best pipeline or to help adapt and optimize the parametrization of the tested pipelines.

At the moment, ToTem’s core function is to efficiently trigger many pipeline configurations and streamline their benchmarking. However, the optimization process itself is not fully automated. Selecting tools and their parameter ranges needs to be done manually, according to the particular data type and thus, this task relies mostly on the knowhow of an experienced user. The primary objective for future development is to provide the option of optimizing the pipeline settings automatically using more complex machine learning algorithms. Implementation will be based on the results collection, mainly from the optimization of pipelines for a specific data type, which can be detected based on their quality control. The data will be anonymized and transformed for the purposes of machine learning applications, which will both select candidates for optimization settings and also select configurations suitable for a specific data type’s routine analysis. Routine analysis results could eventually be used for benchmarking if the user provides feedback. We are also considering installing ToTem using a docker image.

## Conclusion

NGS data analysis workflow quality is significantly affected by the selection of tools and their respective parameters. In this study we present ToTem, a tool enabling the integration of a broad variety of tools and pipelines and their automatic optimization based on benchmarking results controlled through efficient analysis management.

We demonstrated ToTem’s usefulness in increasing the performance of variant calling in two distinct NGS experiments. In the case of somatic variant detection on ultra-deep TGS data, we reached a 2.36-fold improvement in F-measure compared to best performing variant caller’s default settings. In the case of germline variant calling using WGS data, we were able to discover 123,716 additional true positive variants than GATK HaplotypeCaller’s default settings, among those 147 were coding and 70 non-synonymous and of likely functional importance.

## Availability and requirements

**Project name:** ToTem

**Project home page:** https://totem.software

**Operating system(s):** Platform independent

**Programming language:** Java, PHP, MySQL

**Other requirements:** No

**License:** Free for academic use.

**Any restrictions to use by non-academics:** License needed.

## Additional files

Additional file 1: ToTem’s technical documentation. ToTem’s technical documentation describes the technical details of ToTem. (PDF 1464 kb)

Additional file 2: Pseudo code for the Little profet algorithm. The pseudo code describes the general principles of Little Profet algorithm. (TXT 9 kb)

Additional file 3: Material and details of pipeline configurations. The document describes in detail the material and pipeline configurations used in the study. (DOCX 48 kb)

Additional file 4: Detailed performance report generated by Little Profet. The detailed report describing pipeline performance including different over-fitting correction metrics generated by LP. These data were generated as a part of TGS experiment. (XLS 90 kb)

Additional file 5: Performance comparison of 2 variant callers with default and optimized pipelines applied on WGS dataset. The difference between the best pipeline for every tool and the default settings. These data were generated as a part of WGS experiment. (XLSX 14 kb)

## Abbreviations

CLL: Chronic lymphocytic leukemia
CPU: Central processing unit
DSD: Dataset standard deviation
FN: False negative
FP: False positive
GIAB: Genome in a Bottle
GUI: Graphical user interface
HC: High confidence
InDel: Insertion or deletion
LP: Little Profet
MPN: Myeloproliferative neoplasm
NGS: Next generation sequencing
NIST: The National Institute of Standards and Technology
RAM: Random-access memory
SMSD: Sample mix standard deviation
SNV: Single nucleotide variant
TGS: Targeted gene
TP: True positive
UG: GATK UnifiedGenotyper
VAF: Variant allele frequency
VQSR: Variant Quality Score Recalibration
WES: Whole exome sequencing
WGS: Whole genome sequencing
