# Supplementary material for: ToTem: a tool for variant calling pipeline optimization
Source: BMC Bioinformatics. 2018 Jun 26;19:243. doi: 10.1186/s12859-018-2227-x (PMC6020218; doi:10.1186/s12859-018-2227-x)
Supplement: Supplementary file 1 — ToTem’s technical documentation. ToTem’s technical documentation describes the technical details of ToTem. (PDF 1464 kb) [file 12859_2018_2227_MOESM1_ESM.pdf]

Supplementary Information

Technical documentation

Table of Contents

Table of Contents **1**

Workspace **1**

ToTem configuration **2**

- Pipeline & process configuration 2
- Column sets 2
- Reference variant calls 4
- Datasets 4
- Regions of interest (ROI) 4
- Other files 4
- File size limits 4

ToTem execution **5**

Pipeline benchmarking by Little Profet (LP) **5**

- LP configuration 5
- LP results section 1 – Pipelines sorted by F-measure 6
- LP results section 2 – overfitting correction 7
- LP results part 3 – best performing pipelines and intersection of the results 7

ToTem's filtering tool **9**

- Filter configuration part1 9
- Filter configuration part2 9
- Filter results 9

Tools **12**

References **12**

Workspace

During the user profile registration (using email and password), ToTem creates a workspace where all the pipeline configurations, datasets, references, column sets and other data are saved. The workspace is automatically loaded when users log in.

In the next sections, we present ToTem's concept and functions which can be divided into 4 step-wise areas:

- pipeline configuration
- pipeline execution
- pipeline benchmarking and detailed analysis of the results
- additional tools and functions.

ToTem configuration

As an example for all the configuration step, we recommend uploading default data during user registration.

Pipeline & process configuration

The core principle of pipeline optimization in ToTem is to automatically test pipeline performance for all the parameter combinations in a user set ranges.

Each pipeline {#1, Figure S1} is defined as serially linked "processes" {#2, Figure S1}. Each process can execute one or more tools, functions or code. Each pipeline's individual processes are automatically linked - meaning the output of the completed process becomes the input for the next one. Process configuration is described in the next section. As the pipelines can start from any phase, the pipelines' input format is arbitrary (usually fastq, bam, mpileup or vcf). The final output of the pipeline must meet the demands of the tools used in the next step:

- Little Profet (LP) **requires** tables in **tab separated format (e.g. tsv)** with tab delimited column headers (CHROM, POS, REF, ALT) in the first row and containing a list of normalized variants. In cases of more variant alleles in genomic position, each allele should by written in its own row (e.g. use function vcfbreakmulti from vcflib [1]).
- Application of **ToTem's filtering tool** is not restricted to any column header.
- In both cases, the column of interest and their delimiters need to be set during "Column sets" configuration.

Process configuration

For each process needs to be defined:

- name {#3, Figure S1}
- CLI call launching the program {#4, Figure S1}
- input file extension {#5, Figure S1}
- output file extension {#6, Figure S1}
- **template** {#7, Figure S1}
- **parameters** to be tested (optional) {#8, Figure S1}

Processes are constructed based on **template scripts.**
Template script represents a **bash script code with special syntax to include placeholders for automatic testing**.
During pipeline generation, placeholders are replaced by appropriate values (number, text, etc.).
Template scripts are specifically defined for each tool and can also contain ustom code and functions.
Such a script needs to be preceded by an **"echo"** command to be correctly created.

ToTem template scripts use several **[placeholders]** for:

- command line call [cli_call] {#8, Figure S1} launching the tool (mandatory)
- output file extension [output_files_ext] {#9, Figure S1} (mandatory)
- **parameters** [params] {#10, Figure S1} dedicated to inserting the tested parameters to be optimized. Each parameter can be represented simply by their presence or absence, one value, more values, interval or even mathematical functions. Parameter ranges can be easily set through GUI.

As an option, the user can take advantage of other placeholders defined manually during configuration or automatically assigned by ToTem:

- [my_files_dir] {#11, Figure S1} – path to the files uploaded by the user in the section "Your files"
- [dataset_dir] – path to the files uploaded by the user in the section "Datasets"; using this placeholder doesn't require a definition of a specific dataset - datasets are browsed dynamically and appropriate files are selected automatically based on their name. This can be utilized for the example during the GATK variant annotation or variant quality score recalibration (VQSR) [2]
- [input_file_name] {#12, Figure S1} – path to the input file (assigned automatically)
- [output_files_dir] {#13, Figure S1} – path to the output directory (assigned automatically).

Placeholder can be used only in template definition, not for defining the parameters!

Each process can be easily copied with its sub-processes into another category or deleted by **"drag and drop"**.

Column sets

"Column sets" represent columns of interest selected from the tables of results generated by the pipelines. These columns are then imported into ToTem's internal database for further evaluation.

Each column set is defined by:

- Name
- Column delimiter
- Columns

For each column are defined:

- DB column name (column name created in ToTem's database)
- File column name (column name in the file of the results produced by pipeline)
- Type (data type)
- Size (number of letters or digits)
- Default filter type (no default, enumerated value or range)

By default, ToTem includes column sets predefined for:

- tsv tables dedicated to benchmarking by Little Profet method (CHROM, POS, REF, ALT, INFO columns)
- output from RTG Tools and hap.py [3, 4]dedicated to analysis by ToTem's filtering tool (Type, Subtype, Subset, Filter, Genotype, METRIC.Precision, METRIC.Recall, METRIC.F1_Score)

A practical example of column set configuration is provided in a step-by-step tutorial on totem.software web pages.

Figure S1 - Process configuration page

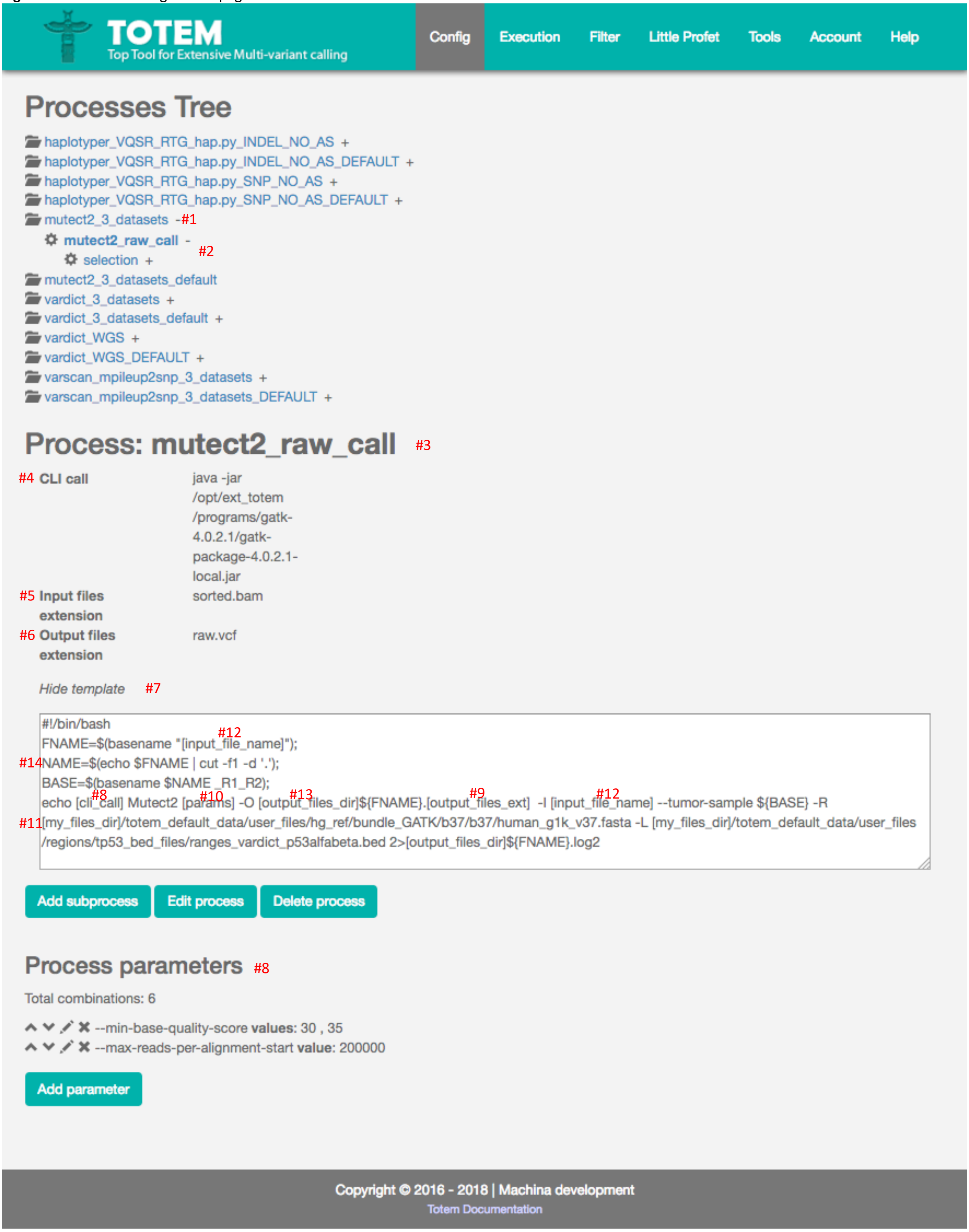

Figure S1 describes the Process configuration page with emphasis on explaining the positioning of placeholders in the template script allowing automatic testing of many pipeline configurations generated based on different parameter values defined in the lower part of figure {#8, Figure S1}. In this particular case, 6 pipelines will be generated. Each parameter is also set to be tested for its default values which are hidden in this particular

case, and visible only during parameter configuration. The core principle of the script generation is to replace the placeholders for their real values, defined during configuration or assigned automatically by ToTem. Variables {#14, Figure S1} used in the script for file name modifications are defined before the script itself, which needs to be preceded by "echo".
A detailed example of process configuration is described in a step-by-step video tutorial on the totem.software web page.

Reference variant calls

"Reference variant calls" represent ground truth variants used for pipeline benchmarking; in the case of ToTem, defined exclusively for the **"Little Profet"** method.
Ground truth variants used by **RTG Tools and hap.py** are defined in an appropriate process configuration as a path to a vcf file and with no need to upload them in this section.

Reference variant call should thus look like:

- the **list of ground truth variants** which can be imported as tab delimited file (CHROM, POS, REF, ALT and SAMPLE columns headers are mandatory). The sample name is defined as the first part of the file name used as an input for pipeline optimization (as a separator "." is used). Such variants are used by the LP algorithm and needs to be imported via ToTem's GUI. Reference variant calls can also be added one by one manually.
- **empty reference** is used by the pipelines including a benchmarking step, e.g. GIAB pipeline using RTG Tools and hap.py.

A practical example of "Reference variant calls" configuration is provided in a step-by-step tutorial on the totem.software web pages.

Datasets

Datasets represent raw input data (usually fastq, bam, mpileup, vcf and their indexes) uploaded using GUI and used for pipeline optimization. Data are stored in the user's workspace and accessible using placeholder **[dataset_dir]**.

Practical example of "Datasets" configuration is provided in a step-by-step tutorial on the totem.software web pages.

Regions of interest (ROI)

ROI represents genomic ranges in which the variants detected by each pipeline are compared to the "Reference variant calls".

- ROIs configured in this section are used exclusively by the "Little Profet" benchmarking method and can be imported as a bed file or added one by one.
- ROI used for e.g. GIAB benchmarking approach are uploaded into "Other files" and the path to them should be defined in the configuration of appropriate processes (RTG Tools and hap.py).

A practical example of using ROI is provided in a step-by-step tutorial on the totem.software web pages.

Other files

"Other files" represent the files necessary to run the pipelines (**bed files, stratification files, reference sequences, etc**.). These files can be imported into ToTem via GUI and are saved into ToTem's workspace. These data are available using a **[my_files_dir]** placeholder.
Human genome reference GRCH37 is available under "[my_files_dir]/totem_default_data/".

File size limits

The limits for file size are currently set to:
5000 MB per file uploaded into "Other files"
1000 MB per file uploaded into "Reference variant calls"
5000 MB per file uploaded into "Datasets"
100 MB per file uploaded into "Regions of interest".

ToTem execution

When the analysis is started, ToTem creates **all possible pipelines** within the pre-set parameter ranges and executes them on the attached computational server. All the combined settings **are executed in parallel,** limited by a defined **maximal number of threads**. The parallelization, resource control and asynchronous communication with the application server is managed by ToTem's backend. The results are imported into ToTem's internal database for consecutive evaluation and benchmarking.

One or multiple pipelines or even only their parts (selected processes) are run on **selected datasets**.

The **execution is divided into 3** parts which can be run separately:

- **generation of every possible configuration** of selected pipelines

- **execution of the pipelines parallel**. Parallelization can be restricted in the ToTem's GUI by the number of threads; one thread = one configuration; the number of threads must be considered according to the hardware requirements of the pipeline.

- **importing of the results into the ToTem's database** for further evaluation performed by Little Profet or ToTem's filtering tool (both approaches require selection of a predefined column set).

Running, waiting and finished processes can be displayed by "**show process list**".

A practical example of ToTem execution is provided in a step-by-step tutorial on the totem.software web pages.

Pipeline benchmarking by Little Profet (LP)

Little Profet is ToTem's **genuine benchmarking method which compares variant calls generated by tested pipelines to the gold standard reference variant call set. LP calculates standard quality metrics (precision, recall and F-measure) and most importantly - reproducibility of each quality metric, which is the main advantage over the standard GIAB approach**. ToTem thus allows the best pipelines to be selected considering the selected quality metrics and its consistency over multiple data subsets. The LP approach is designed primarily for TGS data harbouring a limited number of sequence variants (SNVs, InDels) and suffering from a high risk of pipeline over-fitting.
LP therefore represents an alternative to the GIAB approach with the added value of taking additional measures to guarantee robust results.

LP benchmarking is based on:

- **comparison of normalized variants** (one variant allele per row) detected by each pipeline configuration to the ground truth variants in regions of interest **and inferred precision, recall and F-measure**.
- **over-fitting correction utilizing cross validation approaches that penalize the precision, recall and F-measure scores based on results variation over different data subsets.** The assumption is that the pipelines showing the least variability of results among data subsets will also prove to be more robust when applied to unknown data.

The reproducibility is calculated from all the samples (>3) going into the analysis, while a repeated (number of repeats = ½ of samples) random sub-sampling (number of samples in one sampling group = ½ of samples) validation is performed to estimate the sub-sampling standard deviation (SMSD) of the validation results for individual performance quality metrics (precision, recall and F-measure). The reproducibility may also be inferred from the min/max values for a given performance quality measure calculated for each sub-sampling group. If multiple distinct data sets are provided (at least 2), standard deviation between the selected data set results (DSD) can be used to assess reproducibility as well.
Additionally, to improve the precision and consistency of variant detection, the intersection of the results from each pair of the 10 best performing pipelines (5 pipelines with higher precision, 5 with higher recall) is done by default. The detailed information about pipeline performance including over-fitting correction can be exported to an Excel file for further evaluations.

Source code of this method is available as Additional file 2 and on totem.software web pages.

LP configuration

To run LP, the user needs to select:

- regions of interest
- variant type
- datasets (their variant calling results must be already imported into ToTem's database)
- pipelines

The analysis is done for **all configurations for a selected pipeline** by default, but **can be restricted** to specific pipelines by defining their **"settings IDs"** separated by "," into an appropriate box. LP uses normalized variant tables (normalization and vcf to table format conversion should be the part of the pipeline) as an input, with mandatory tab delimited headers (CHROM, POS, REF, ALT). The analysis is restricted to selected ROI.

A practical example of using Little Profet is provided in a step-by-step tutorial on the totem.software web pages.

LP results section 1 – Pipelines sorted by F-measure

As for the results, LP provides several graphs and tables. The first section of the LP results can be typified as Figure S2, which is divided into 2 parts:
The first part represents an **interactive pipeline table sorted according to F-measure**. Going from the left:

- Checking the select box {#1, Figure S2} has several functions:
  - **highlighting** (enlarging the dot) pipelines in associated LP graphs {#2, Figure S2}
  - selecting pipelines (champions) for the pair-wise **intersection** of the results of each pipeline {#1, Figure S4}.
- Setting ID = ID of the pipeline;
- Rows = number of records;
- TP = number of true positives (hyper-link for viewing the details);
- FP = number of false positives (hyper-link for viewing the details);
- FN = number of false negatives (hyper-link for viewing the details);
- RMM = number of reference mismatches between pipelines results and ground truth reference variant calls;
- Recall, Precision and F-measure are calculated based on all samples from selected datasets;
- ToTem F-measure SMSD (corrected F-measure) = F-measure – SMSD;
- SMSD = SD based on repeated random sub-sampling;
- DSD = SD deviation between datasets;
- "show pipeline" and "show details" {#3, Figure S2} provides detailed info about pipeline settings, TP, FP, FN, recall, precision, F-measure and reproducibility.

The lower part of the figure represents an **interactive graphical interpretation** of the table above.

- Each dot corresponds to recall (X-axis) and precision (Y-axis) for 1 pipeline configuration. Precision and recall are calculated based on all samples for all three variant callers – Variant Caller 1, Variant Caller 2 and Variant Caller 3 which are distinguished by color coding. Each variant caller is represented by 2 best performing pipelines (1 with higher precision, 1 with higher recall) and default configuration which were selected using "Setting ID" {#4, Figure S2}. ToTem also offers interactive information when pointing the dot {#5, Figure S2}.

Figure S2 – LP results: Pipelines sorted by F-measure

Setting IDs

#4 85433, 85543, 84831, 84846, 85547, 324:

Run!

Results sorted by F-measure

#1	Setting ID	Rows	TP	FP	FN	RMM	Recall	Precision	F-measure	Totem F-measure SMSD	DSD	SMSD	#3	
☑	32414	1026	901	125	111	0	0.8903	0.8782	0.8842	0.8722	0.0159	0.012	show pipeline	show details
☑	32417	985	877	108	135	0	0.8666	0.8904	0.8783	0.8626	0.0145	0.0157	show pipeline	show details
☐	84846	851	755	95	256	1	0.7468	0.8882	0.8114	0.7989	0.0353	0.0125	show pipeline	show details
☐	84831	1910	898	1011	113	1	0.8882	0.4704	0.6151	0.5881	0.0974	0.027	show pipeline	show details
☐	85547	285	270	15	742	0	0.2668	0.9474	0.4163	0.3824	0.1301	0.034	show pipeline	show details
☐	85433	271	260	11	752	0	0.2569	0.9594	0.4053	0.3689	0.1425	0.0364	show pipeline	show details
☐	85543	306	257	49	755	0	0.2540	0.8399	0.3900	0.357	0.1353	0.033	show pipeline	show details
☐	25728	236	234	2	778	0	0.2312	0.9915	0.3750	0.3412	0.1393	0.0338	show pipeline	show details

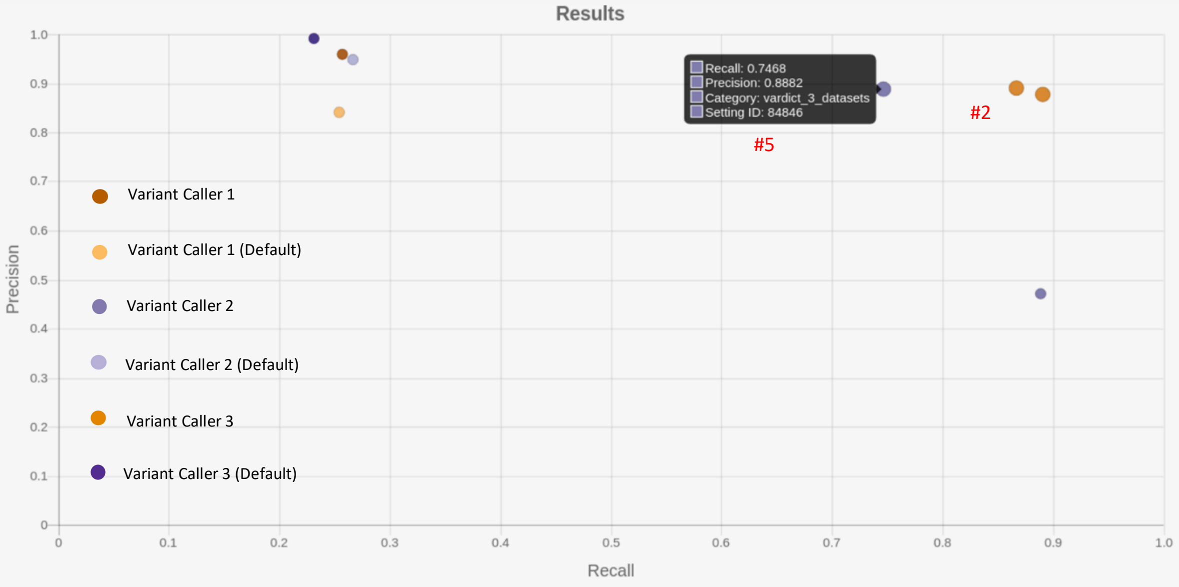

LP results section 2 – overfitting correction

The second section of the LP results provides a **visualization** of pipelines' **reproducibility** and is divided into 2 parts:

- **"Sniper deviation"** graph where each dot represents an **arithmetic mean** of **recall** (X-axis) and **precision** (Y-axis) for 1 pipeline configuration calculated based on **repeated random sub-sampling** of 3 datasets. The size of the line on the cross's horizontal axis represents **2SMSD** for **recall**, the cross's **vertical axis represents 2SMSD** for **precision**. Each variant caller (Variant Caller 1, Variant Caller 2 and Variant Caller 3) is represented by the 2 best performing pipeline configurations (1 with higher precision, 1 with higher recall) and 1 with default settings. Highlighting the 2 best performing pipelines in the upper right corner {#1, Figure S3} is the effect of selecting the checkbox in Figure S2. The interactive information about the pipeline can easily be visualized when pointing the dot.
- **"Sniper distortion"** graph showing 2 best performing pipeline configurations (1 with higher precision, 1 with higher recall) and 1 with default settings for Variant Caller 1, Variant Caller 2 and Variant Caller 3. Each dot represents an **arithmetic mean** of **recall** (X-axis) and **precision** (Y-axis) for 1 pipeline calculated based on **repeated random sub-sampling** of 3 datasets. The **borders** of the **horizontal axis** on the cross represent the **minimum** and **maximum** of each pipeline's **recall**. **Vertical axis** of the cross represents the **interval** of pipeline's **precision**. An interactive info is provided when pointing the dot. Highlighting the 2 best performing pipelines in the upper right corner {#2, Figure S3}is the effect of selecting the checkbox in Figure S2.

Figure S3 – Little Profet results: over-fitting correction and reproducibility interpretation.

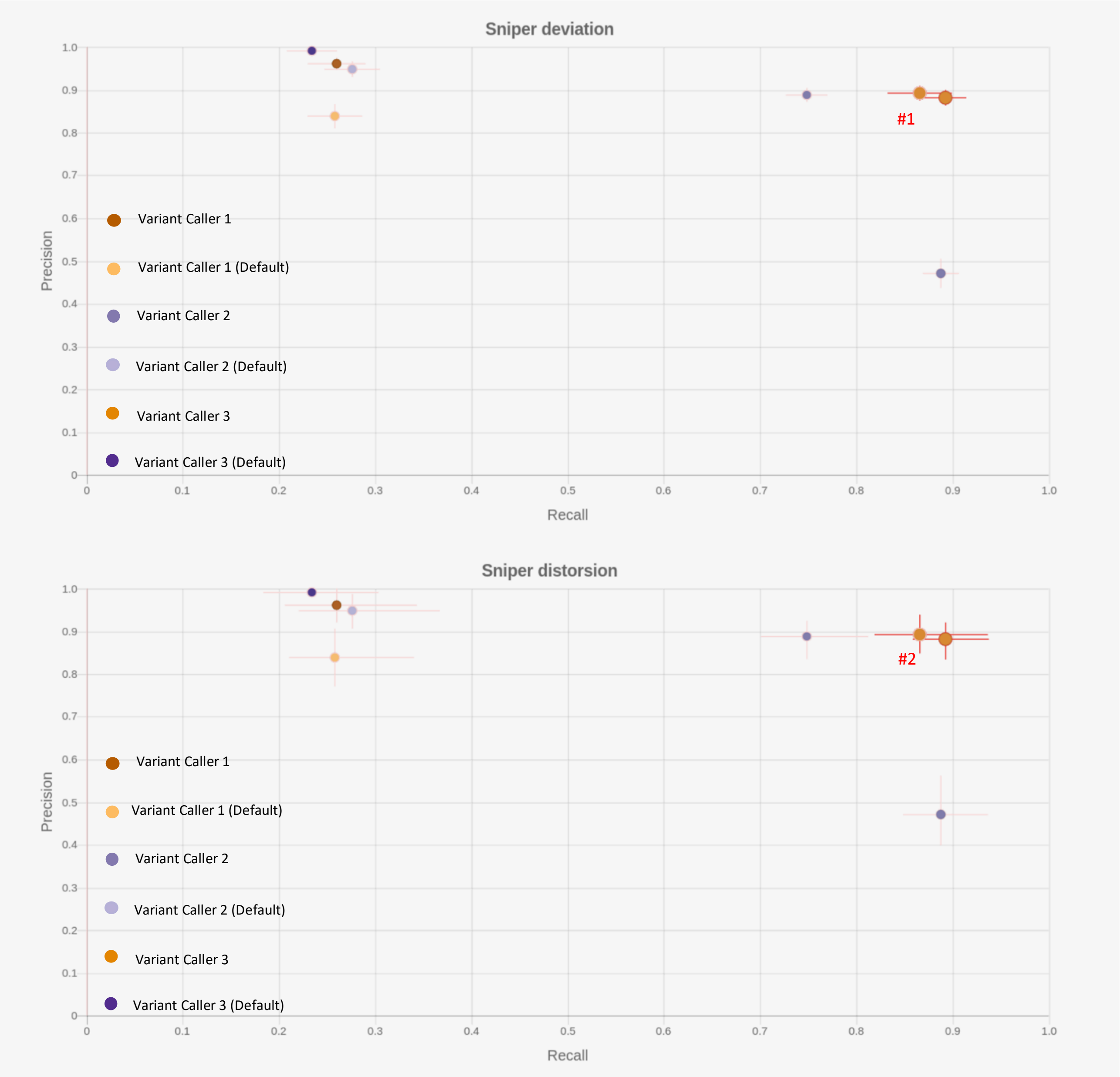

LP results part 3 – best performing pipelines and intersection of the results

The third part of the LP results (Figure S4) shows 2 tables listing the best performing configurations called **"Champions"** {#1, Figure S4} and the intersection of their results called **"Champions pairs"** {#2, Figure S4}.

Champions represent the best **10 performing pipelines** (5 pipelines with higher precision, 5 with higher recall by default, sorted by F-measure) by default. Champions can also be manually selected using checkbox {#1, Figure S2}.

The table of **Champion pairs** represents an **intersection** of the results of all Champions' pairs aiming to improve the variant detection's precision.

The detailed results generated by LP can be exported into an **excel table** using “Export to Excel document” and used for further evaluation {#3, Figure S4}. The “Champions” and “Champions pairs” can be visualized via graphs similarly like pipelines in Figure S2 and Figure S3 {#4, Figure S4 and #5, Figure S4}.

The meaning of the column headers is the same for both tables:

- Setting ID = ID of the pipeline;
- Rows = number of records;
- TP = number of true positives (hyper-link for viewing the details);
- FP = number of false positives (hyper-link for viewing the details);
- FN = number of false negatives (hyper-link for viewing the details);
- RMM = number of reference mismatches between pipelines results and ground truth reference variants;
- Recall, Precision and F-measure are calculated based on all samples from selected datasets;
- ToTem F-measure SMSD= F-measure – SMSD;
- SMSD = repeated random sub-sampling based SD;
- DSD = SD deviation between datasets;
- “show pipeline” and “show details” provides detailed info about pipeline settings, TP, FP, FN, recall, precision, F-measure.

Figure S4 - Little Profet results: best performing pipelines and intersection of their results

#1 Champions

Setting ID	Rows	TP	FP	FN	RMM	Recall	Precision	F-measure	Totem F-measure SMSD	DSD	SMSD		
32414	1026	901	125	111	0	0.8903	0.8782	0.8842	0.8722	0.0159	0.012	show pipeline	show details
32417	985	877	108	135	0	0.8666	0.8904	0.8783	0.8626	0.0145	0.0157	show pipeline	show details
84831	1910	898	1011	113	1	0.8882	0.4704	0.6151	0.5881	0.0974	0.027	show pipeline	show details
84846	851	755	95	256	1	0.7468	0.8882	0.8114	0.7989	0.0353	0.0125	show pipeline	show details
85547	285	270	15	742	0	0.2668	0.9474	0.4163	0.3824	0.1301	0.034	show pipeline	show details
85433	271	260	11	752	0	0.2569	0.9594	0.4053	0.3689	0.1425	0.0364	show pipeline	show details
85543	306	257	49	755	0	0.2540	0.8399	0.3900	0.357	0.1353	0.033	show pipeline	show details
25728	236	234	2	778	0	0.2312	0.9915	0.3750	0.3412	0.1393	0.0338	show pipeline	show details

#4 Show graphs

#2 Champion pairs

Setting IDs	Rows	TP	FP	FN	Recall	Precision	F-measure		
32414<br>32417	985	877	108	135	0.8666	0.8904	0.8783	show P1 pipeline<br>show P2 pipeline	show P1 details<br>show P2 details
32414<br>84831	931	838	93	174	0.8281	0.9001	0.8626	show P1 pipeline<br>show P2 pipeline	show P1 details<br>show P2 details
32417<br>84831	901	817	84	195	0.8073	0.9068	0.8542	show P1 pipeline<br>show P2 pipeline	show P1 details<br>show P2 details
32414<br>84846	776	745	31	267	0.7362	0.9601	0.8333	show P1 pipeline<br>show P2 pipeline	show P1 details<br>show P2 details
32417<br>84846	758	731	27	281	0.7223	0.9644	0.8260	show P1 pipeline<br>show P2 pipeline	show P1 details<br>show P2 details
84831<br>84846	818	738	79	273	0.7300	0.9033	0.8074	show P1 pipeline<br>show P2 pipeline	show P1 details<br>show P2 details
32414<br>85547	269	268	1	744	0.2648	0.9963	0.4184	show P1 pipeline<br>show P2 pipeline	show P1 details<br>show P2 details
32417<br>85547	269	268	1	744	0.2648	0.9963	0.4184	show P1 pipeline<br>show P2 pipeline	show P1 details<br>show P2 details
32414<br>85433	259	258	1	754	0.2549	0.9961	0.4060	show P1 pipeline<br>show P2 pipeline	show P1 details<br>show P2 details
32417<br>85433	259	258	1	754	0.2549	0.9961	0.4060	show P1 pipeline<br>show P2 pipeline	show P1 details<br>show P2 details

Show all records

#5 Show graph

Export to Excel document #3

Copyright © 2016 - 2017 | Machina development
Totem Wiki

ToTem's filtering tool

ToTem's filtering tool is dedicated to filtering (stratified) performance reports generated by external benchmarking tools, which are incorporated as a final part of the tested analytical pipelines. This allows testing of many combinations of parameters and **simple selection** of settings that produce **the best results considering e.g. selected quality metrics**, variant type and region of interest (variables depend on the report). This approach is particularly useful for optimizing the pipeline for **WGS or WES and also TGS**.

In the case of this study, we use ToTem's filtering tool to analyze pipelines benchmarked by the GIAB approach, which combines RTG Tools and hap.py. This approach is best suited to variant calling pipelines handling the data which might harbour complex variants and require variant and region stratification, e. g. WGS data. RTG Tools use complex matching algorithms and standardized counting applied for variant normalization and comparison to the ground truth. hap.py is applied for variant and region annotation/stratification. These tools serve as reference implementations of the benchmarking standards agreed upon by the ga4gh data working group [5]. In ToTem's pipeline optimization concept, RTG Tools and hap.py are used to be a final part of the pipeline providing as a result, a regionally stratified performance (precision, recall, F-measure, etc.) report for several variant types.

The performance reports are imported into the internal database and processed by ToTem's filtering tool, allowing easy selection of an optimal pipeline based on the user's needs and priorities. This could be extremely useful while ranking the pipelines for a specific variant type, e.g. single nucleotide variant (SNV) versus insertion or deletion (InDel), variant calling filters and/or specific regions of the genome low-mappability regions, low-complexity regions, AT-rich regions, homopolymers, etc. described as significantly influencing the variant calling performance [6]. The complete list of filtered results describing performance qualities for the selected variant type and region for all the pipelines can be exported into a csv table for deeper evaluation.

ToTem's filtering tool utility is not only restricted to the GIAB approach but can be applied also on other formats of tables describing pipeline performance. The specific format, e.g. column names, column separator, needs to be set through ToTem GUI interface before importing pipeline results into the database.

A practical example of using Totem's filtering tool is provided in a step-by-step tutorial on the totem.software web pages.

Filter configuration part1

In the first step of filtering, the user selects:

- datasets {#1, Figure S5}
- categories {#2, Figure S5}
- columns and columns' data type (enum, range, blank){#3, Figure S5}.

Filter configuration part2

In the second configuration step, the user specifies the selected filters {#3, Figure S5} in the form of **values** {#1, Figure S6} or **ranges** {{#2, Figure S6}.
The lower part of the window shows the example of pre-filtered data corresponding to the raw unfiltered results {#3, Figure S6}. The shown data are restricted to columns defined during "column set" configuration and "Filter configuration part 1".
The example of unfiltered results can be exported into a csv table using the "Download as csv" button {#4, Figure S6}.
"Show pipeline" link provides information about pipeline settings.

Filter results

The results (based on the output of hap.py) are provided in the form of a table as typified in Figure S7.
The upper part of the window corresponds to the **applied filters** which are still configurable {#1, Figure S7}.
The lower part of the window represents the **columns of interest** defined before (Column Sets) and **rows fitting the applied filters**. Only pipelines having specified recall are on the list. In this particular example, precision, recall and F-measure for pipelines detecting SNPs of all variant subtypes, regional subsets and genotypes which pass the filters or filtered out are described {#2, Figure S7}. The **complete list of filtered results** can be exported into a **csv table**. For further evaluation using the "Download as csv" button {#3, Figure S7}. The information about pipeline settings is provided via "Show pipeline" link {#4, Figure S7}.

Figure S5 – Main page of ToTem's Filtering tool

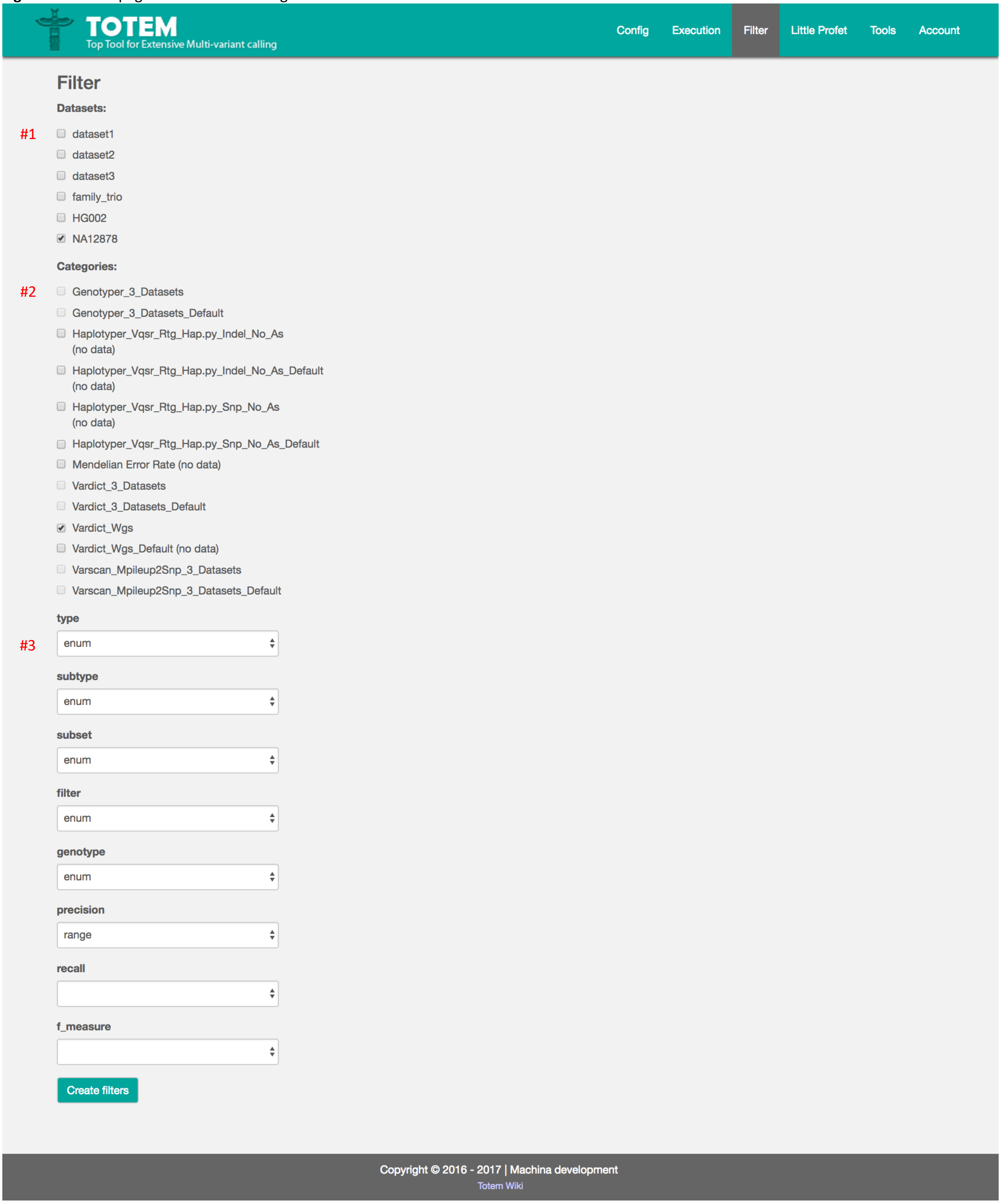

Figure S6 – ToTem's Filtering tool: setting the filters

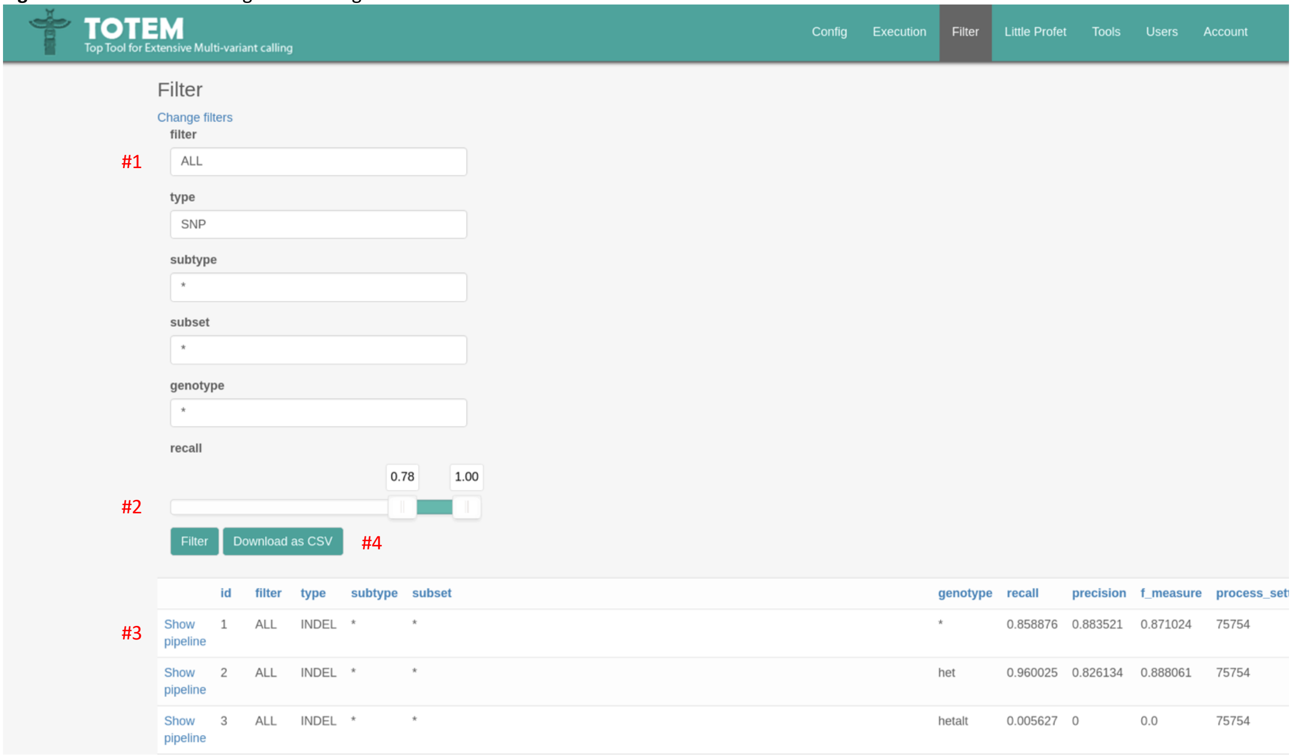

Figure S7 – ToTem' Filtering tool: results

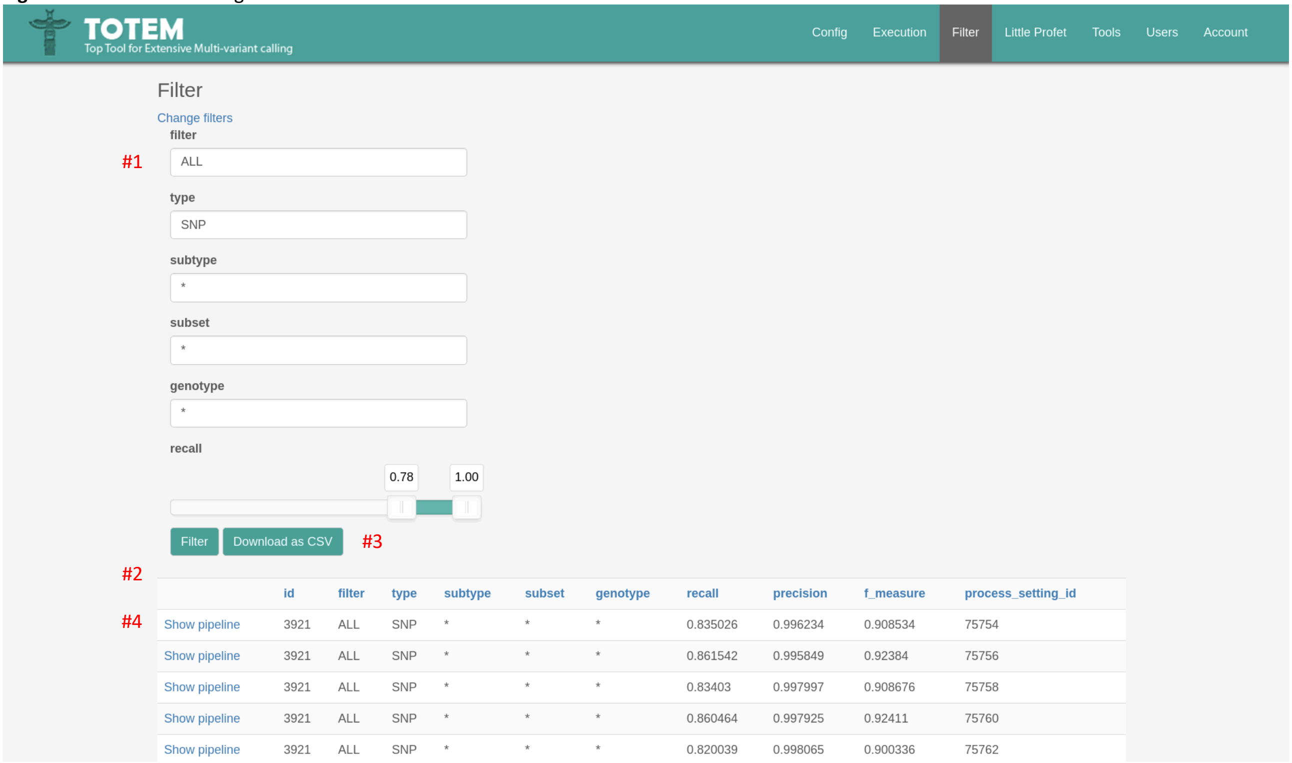

Tools

ToTem provides several functions to facilitate its handling, maintenance and backup. All of the functions are available for administrative users and only some of them are available for standard users.
ToTem includes functions for:

- saving/exporting ToTem's databases with configurations and results (admin)
- managing a cache (admin)
- removing unused results (admin)
- updating (admin)
- combining outputs of multiple variant callers (VcfGlue)
- providing detailed information about TP, FP and FN (information taken from ground truth variants set or from the output of variant callers)
- importing/exporting configurations
- monitoring finished, running and waiting processes
- managing reference variant calls
- managing datasets
- managing regions of interest
- managing "Other files"
- access through API through "Account" page.

References

1. vcflib: a simple C++ library for parsing and manipulating VCF files, + many command-line utilities. C++. vcflib; 2017. https://github.com/vcflib/vcflib. Accessed 22 Dec 2017.

2. DePristo MA, Banks E, Poplin RE, Garimella KV, Maguire JR, Hartl C, et al. A framework for variation discovery and genotyping using next-generation DNA sequencing data. Nat Genet. 2011;43:491–8.

3. rtg-tools: RTG Tools: Utilities for accurate VCF comparison and manipulation. Java. Real Time Genomics; 2017. https://github.com/RealTimeGenomics/rtg-tools. Accessed 18 Dec 2017.

4. hap.py: Haplotype VCF comparison tools. C++. Illumina; 2017. https://github.com/Illumina/hap.py. Accessed 18 Dec 2017.

5. ga4gh GitHub. HTML. Global Alliance for Genomics and Health; 2017. https://github.com/ga4gh/benchmarking-tools. Accessed 22 Dec 2017.

6. Popitsch N, WGS500 Consortium, Schuh A, Taylor JC. ReliableGenome: annotation of genomic regions with high/low variant calling concordance. Bioinforma Oxf Engl. 2017;33:155–60.
