# Supplementary material for: ToTem: a tool for variant calling pipeline optimization
Source: BMC Bioinformatics. 2018 Jun 26;19:243. doi: 10.1186/s12859-018-2227-x (PMC6020218; doi:10.1186/s12859-018-2227-x)
Supplement: Supplementary file 3 — Material and details of pipeline configurations. The document describes in detail the material and pipeline configurations used in the study. (DOCX 48 kb) [file 12859_2018_2227_MOESM3_ESM.docx]

*Supplementary Information*

*Material and pipeline configurations*

Table of Contents

[Material 2](#_Toc515302537)

[TGS datasets 2](#_Toc515302538)

[TGS1 dataset 2](#_Toc515302539)

[TGS2 dataset 2](#_Toc515302540)

[TGS3 dataset 2](#_Toc515302541)

[WGS dataset 2](#_Toc515302542)

[Used tools 3](#_Toc515302543)

[TGS ultra-deep sequencing analysis 3](#_Toc515302544)

[Pre-processing 3](#_Toc515302545)

[Raw variant calling 3](#_Toc515302546)

[Mutect2 3](#_Toc515302547)

[VarDictJava 3](#_Toc515302548)

[VarScan2 4](#_Toc515302549)

[Hard filter optimization 4](#_Toc515302550)

[VarDict 4](#_Toc515302551)

[VarScan2 4](#_Toc515302552)

[Post-processing 4](#_Toc515302553)

[GATK - variant selection 4](#_Toc515302554)

[vcflib - variant normalization and conversion into tsv file 5](#_Toc515302555)

[WGS analysis 5](#_Toc515302556)

[Preprocessing 5](#_Toc515302557)

[Picard tools - Remove duplicates 5](#_Toc515302558)

[Picard tools - Add Read Groups 5](#_Toc515302559)

[GATK Base Quality Score Recalibration step1 5](#_Toc515302560)

[GATK Base Quality Score Recalibration step2 5](#_Toc515302561)

[Picard tools - Downsample BAM 5](#_Toc515302562)

[Raw variant calling 5](#_Toc515302563)

[GATK HaplotypeCaller 5](#_Toc515302564)

[VarDict 6](#_Toc515302565)

[Edit header of ground truth vcf 6](#_Toc515302566)

[Create intersect of HC intervals between both individuals 6](#_Toc515302567)

[Variant filtering optimization 6](#_Toc515302568)

[GATK HaplotypeCaller VQSR SNV step1 6](#_Toc515302569)

[GATK HaplotypeCaller VQSR SNV step2 7](#_Toc515302570)

[GATK HaplotypeCaller VQSR INDEL step1 7](#_Toc515302571)

[GATK HaplotypeCaller VQSR INDEL step2 8](#_Toc515302572)

[VarDict 8](#_Toc515302573)

[Post-processing 8](#_Toc515302574)

[Bgzip - vcf compression 8](#_Toc515302575)

[RTG Tools - comparison of filtered variants to the ground truth 8](#_Toc515302576)

[hap.py - variant type and region stratification 9](#_Toc515302577)

[References 9](#_Toc515302578)

Material

Ground truth reference material allows measurement of sequencing performance and bioinformatics methods, including false positive and false negative rates. Reference material usually consists of raw sequencing data or alignments and associated set of validated variants. To showcase the functionality of ToTem, 3 datasets representing TGS data and WGS data from 2 individuals were used.

TGS datasets

Benchmark material for targeted ultra-deep sequencing (exons 2 – 11 of *TP53* gene) was divided into 3 datasets of samples based on differences in diagnosis, verification status, and mutation load. Datasets were analyzed by different bioinformatics approaches. This was purposefully used to determine the pipelines’ reproducibility.

The whole study and written informed consent obtained from all patients analysed for variant discovery in *TP53* were approved by the Ethical Committee of University Hospital Brno in concordance with the Declaration of Helsinki.

All DNA samples were sequenced with ultra-high coverage (min. coverage depth > 5000x, average depth of coverage approx. 35 000x) using Nextera XT DNA Sample Preparation Kit and MiSeq Reagent Kit v2 (300 cycles) (Illumina, San Diego, CA, USA) on a MiSeq instrument as described previously [1]. Reads’ quality trimming, merging and mapping onto the reference genome (GRCh37) as well as variant calling was done using CLC Genomic Workbench [version 7.5]. Shearwater algorithm from R- package DeepSNV [2] was used as the second variant calling approach.

The minimum variant read count was set to 10. Only variants detected either by both variant calling algorithms or confirmed by a technical or biological replicate were added to the list of candidate ground truth variants. To remove remaining FP, filtering was applied according to VAF present in an in-house database containing all the samples processed in our laboratory. Because an in-house database accumulates false-positive variants specific for the used sequencing platform, sequencer and analysis pipeline, it could be used to identify and remove these FP. All computationally predicted variants were manually checked by expert users and confirmed by biological findings [1, 3]. This approach allowed us to detect variants down to 0.1% VAF.

Only SNV were considered during the analysis. Short InDels were not included in the ground truth set due to their insufficient quantity.

TGS1 dataset

TGS 1 dataset was represented by 355 SNVs detected in 103 samples of patients diagnosed with chronic lymphocytic leukemia (CLL). The dataset represented variants detected in VAF ranging from 0.1-100%. Variant calling was done by CLC Genomic Workbench and Shearwater algorithm. Only variants confirmed by both algorithms or by a biological/technical replicate were taken into account. The dataset should not contain any false positives variants.

TGS2 dataset

TGS 2 dataset consisted of 248 SNVs present in 77 patient samples with myeloproliferative neoplasm (MPN). With the exception of known germline polymorphisms, variants representing low burden sub-clones up to 10% VAF prevailed, as fully expanded (>20%VAF) TP53 mutations are rare in MPN [3]. Only variants detected by CLC Genomic Workbench, confirmed by technical replicates or by an independent sampling were used. The dataset should not contain any false positives variants.

TGS3 dataset

TGS3 dataset was represented by 409 SNVs detected in 40 patient samples with CLL with VAF 0.1-100%. Variant calling was done using CLC Genomic Workbench only and could rarely contain false positives.

WGS dataset

NA12878 and HG002 genomes were analyzed by The Genome in a Bottle Consortium (GIAB), hosted by the National Institute of Standards and Technology (NIST) which is creating reference materials and data for human genome sequencing. The data came from 12 sequencing technologies and several bioinformatics approaches guaranteeing high confidence (HC) of reported variants [4].

Alignments (hs37.d5.300x.bam) for NA12878 and HG002 individuals were downloaded from:

<ftp://ftp-trace.ncbi.nlm.nih.gov/giab/ftp/data/NA12878/NIST_NA12878_HG001_HiSeq_300x/NHGRI_Illumina300X_novoalign_bams/>

and

ftp://ftp-trace.ncbi.nlm.nih.gov/giab/ftp/data/AshkenazimTrio/HG002_NA24385_son/NIST_HiSeq_HG002_Homogeneity-10953946/NHGRI_Illumina300X_AJtrio_novoalign_bams/

Associated ground truth variants, high confidence regions and stratification files [version 3.3.2] were downloaded from:

<ftp://ftp-trace.ncbi.nlm.nih.gov/giab/ftp/release/NA12878_HG001/NISTv3.3.2/GRCh37/>

and

<ftp://ftp-trace.ncbi.nlm.nih.gov/giab/ftp/release/AshkenazimTrio/HG002_NA24385_son/NISTv3.3.2/GRCh37/>

# Used tools

bedtools2 (version 2.19.1) [5]

BWA – MEM (version 0.7.12) [6]

SAMtools (version 1.2) [7]

gatk (version 3.6, v4) [8, 9]

hap.py (version 0.3.3) [10]

Picard Tools (version 2.6.0) [11]

RTG Tools (version 3.7) [12]

Variant Caller 2 (version 1.2) [13]

Variant Caller 3 (version 2.3.9) [14, 15]

vcflib (version 1.0.0) [16]

TGS ultra-deep sequencing analysis

Pre-processing

Demultiplexed sequencing reads were mapped onto the reference genome (GRCh37) using BWA-MEM algorithm with default settings. Conversion from SAM to BAM, sorting and indexing was performed using SAMtools.

Raw variant calling

In the next step, Mutect2, VarDict and VarScan2 tools were optimized for variant calling performance. Only *TP53* exons 2-11 +/- 2 bp (splicing sites) were considered during the analysis. Settings for raw variant calling, hard filtering, (annotations), selection and normalization are described below.

Mutect2

(Total combinations: 36)

**CLI call:** java -jar GenomeAnalysisTK4.jar

**Input files extension:** sorted.bam

**Output files extension:** raw.vcf

**Template:**

#!/bin/bash

FNAME=$(basename "[input_file_name]");

echo [cli_call] -T UnifiedGenotyper [params] -o [output_files_dir]${FNAME}.[output_files_ext] --logging_level ERROR --log_to_file [output_files_dir]${FNAME}.log -I [input_file_name] 2>[output_files_dir]${FNAME}.log2

**Parameters:**

-R value: [human_g1k_v37.fasta]

-L value: [p53_exons+beta_splice2nt.bed]

--sample-ploidy [default, 3]

--min-base-quality-score [default, 30, 35]

--tumor-lod-to-emit [default, 10.0, 15.0]

--max-reads-per-alignment-start [default, 200000]

VarDictJava

(Total combinations: 1)

**CLI call:** VarDictJava/build/install/VarDict/bin/VarDict

**Input files extension:** sorted.bam

**Output files extension:** vardict

**Template:**

#!/bin/bash

FNAME=$(basename "[input_file_name]");

echo [cli_call] [params] -b [input_file_name] ranges_vardict_p53alfabeta.bed | VarDictJava/VarDict/teststrandbias.R > [output_files_dir]${FNAME}.[output_files_ext] 2>[output_files_dir]${FNAME}.log

**Parameters:**

-c value: [1]

-S value: [2]

-E value: [3]

-G value: human_g1k_v37.fasta

-f value: [0.000001]

-Q value: [1]

-P value: [1]

-m value: [15]

-q value: [15]

-th value: [100]

# file ranges_vardict_p53alfabeta.bed consists of exons’ *TP53* exons 2-11 +/- 2 bp (splicing sites) prolonged by 100 bp.

VarScan2

In case of VarScan2, initial raw variant calling was done by SAMtools outside ToTem. The output of raw variant calling was uploaded into ToTem and used as an input for extensive hard filter optimization.

samtools-1.2/samtools mpileup -f human_g1k_v37.fasta -d 999999 -F 0.00001 input.bam > input.bam.mpileup

Hard filter optimization

VarDict

(Total combinations: 192)

**CLI call:** VarDictJava/VarDict/var2vcf_valid.pl

**Input files extension:** vardict

**Output files extension:** vcf

**Template:**

#!/bin/bash

FNAME=$(basename "[input_file_name]");

echo cat [input_file_name] | [cli_call] [params] > [output_files_dir]${FNAME}.[output_files_ext] 2>[output_files_dir]${FNAME}.log

**Parameters:**

-v value: [default, 8]

-c values: [default , 3]

-p values: [default , 10]

-P values: [default , 1]

-q values: [default , 35]

-f values: [default, 0.001 , 0.002]

-m values: [default, 10]

VarScan2

(Total combinations: 54)

**CLI call:** java -jar VarScan.v2.3.9.jar mpileup2snp

**Input files extension:** mpileup

**Output files extension:** varscan.snp.vcf

**Template:**

#!/bin/bash

FNAME=$(basename "[input_file_name]");

echo [cli_call] [input_file_name] [params] > [output_files_dir]${FNAME}.[output_files_ext]

**Parameters:**

--min-var-freq values: [default, 0.001 , 0.002]

--min-avg-qual values: [default , 35]

--min-reads2 [default, 6, 10]

--output-vcf

--p-value values: [default , 0.95]

Post-processing

For all 3 variant callers, only variants passing filters were selected by GATK SelectVariants and then normalized, converted into tsv format by vcflib. Tsv files were uploaded into ToTem’s internal database and further processed by Little Profet benchmarking approach.

GATK - variant selection

(Total combinations: 1)

**CLI call:** java -jar GenomeAnalysisTK.jar

**Input files extension:** [variant_caller_dependent].vcf

**Output files extension:** selected.vcf

**Template:**

#!/bin/bash

FNAME=$(basename "[input_file_name]");

echo [cli_call] -T SelectVariants -R human_g1k_v37.fasta -ef [params] [input_file_name] -o [output_files_dir]${FNAME}.[output_files_ext] --logging_level ERROR --log_to_file [output_files_dir]${FNAME}.log

**Parameters:**

--variant

vcflib - variant normalization and conversion into tsv file

(Total combinations: 1)

**CLI call:** vcflib/bin/breakmulti

**Input files extension:** selected.vcf

**Output files extension:** tsv

**Template:**

#!/bin/bash

FNAME=$(basename "[input_file_name]");

echo [cli_call] [params] [input_file_name] | vcflib/bin/vcfallelicprimitives –k –use-mnps | vcflib/bin/vcf2tsv -g > [output_files_dir]${FNAME}.[output_files_ext] 2>[output_files_dir]${FNAME}.log

**#All the procedures starting with raw variant calling were also performed using default parameters to evaluate the advantage of variant calling optimization.**

WGS analysis

Preprocessing

Read alignments of both individuals downloaded from the GIAB ftp server were firstly preprocessed using the following tools and functions:

HG001.hs37d5.300x.bam was renamed to NA12878.hs37d5.300x.bam

Picard tools - Remove duplicates

java -jar picard.jar MarkDuplicates I=[individual].hs37d5.300x.bam O=[individual].hs37d5.300x.bam.dedup.bam M=[individual].hs37d5.300x.bam.duplicates_report.txt ASSUME_SORTED=TRUE REMOVE_DUPLICATES=TRUE VALIDATION_STRINGENCY=LENIENT CREATE_INDEX=TRUE

Picard tools - Add Read Groups

java -jar picard.jar AddOrReplaceReadGroups I=[individual].hs37d5.300x.bam.dedup.bam O=[individual].hs37d5.300x.bam.dedup.bam.RG.bam RGLB=unknown RGPL=illumina RGPU=unknown RGSM=individual CREATE_INDEX=true

GATK Base Quality Score Recalibration step1

java -Xmx12G -jar GenomeAnalysisTK.jar -R hs37d5.fa -T BaseRecalibrator -I [individual].hs37d5.300x.bam.dedup.bam.RG.bam -o [individual].hs37d5.300x.bam.dedup.bam.RG.bam.recal_data.table -nct 100 -knownSites common_all_20161121.vcf.gz

GATK Base Quality Score Recalibration step2

java -Xmx12G -jar GenomeAnalysisTK.jar -R hs37d5.fa -T PrintReads -I [individual].hs37d5.300x.bam.dedup.bam.RG.bam -o [individual].hs37d5.300x.bam.dedup.bam.RG.bam.BQSR.bam -BQSR [individual].hs37d5.300x.bam.dedup.bam.RG.bam.recal_data.table -nct 50

Picard tools - Downsample BAM

java -jar picard.jar DownsampleSam I= [individual].hs37d5.300x.bam.dedup.bam.RG.BQSR.bam O=[individual].hs37d5.300x.bam.dedup.bam.RG.BQSR.bam.down0.1.bam PROBABILITY=0.1 CREATE_INDEX=true

Raw variant calling

For variant calling optimization, VarDict and GATK HaplotyperCaller followed by VQSR were tested. The initial raw variant calling for both variant callers was performed outside ToTem. The following extensive hard filter (VarDict) and VQSR (GATK HaplotypeCaller) optimization was done in ToTem.

GATK HaplotypeCaller

java -Xmx12G -jar GenomeAnalysisTK.jar -R hs37d5.fa -T HaplotypeCaller -I [individual].hs37d5.300x.bam.dedup.bam.RG.BQSR.bam.down0.1.bam -o [individual].hs37d5.300x.bam.dedup.bam.RG.BQSR.bam.down0.1.bam.haplotyper.raw.01.vcf -U LENIENT_VCF_PROCESSING --logging_level INFO --log_to_file [individual].hs37d5.300x.bam.dedup.bam.RG.BQSR.bam.down0.1.bam.haplotyper.raw.01.vcf.log -nct 1 -dt NONE --dbsnp common_all_20161121.vcf.gz --output_mode EMIT_VARIANTS_ONLY --genotyping_mode DISCOVERY --min_base_quality_score 15 --standard_min_confidence_threshold_for_calling 10 -A AS_StrandOddsRatio -A AS_ReadPosRankSumTest -A AS_RMSMappingQuality -A AS_QualByDepth -A AS_MappingQualityRankSumTest -A AS_MQMateRankSumTest -A AS_InsertSizeRankSum -A AS_InbreedingCoeff -A AS_FisherStrand -A AS_BaseQualityRankSumTest -A VariantType -A TransmissionDisequilibriumTest -A TandemRepeatAnnotator -A StrandOddsRatio -A StrandBiasBySample -A StrandAlleleCountsBySample -A SpanningDeletions -A SnpEff -A SampleList -A ReadPosRankSumTest -A RMSMappingQuality -A QualByDepth -A PossibleDeNovo -A OxoGReadCounts -A NBaseCount -A MappingQualityZeroBySample -A MappingQualityZero -A MappingQualityRankSumTest -A MVLikelihoodRatio -A LowMQ -A LikelihoodRankSumTest -A InbreedingCoeff -A HomopolymerRun -A HardyWeinberg -A HaplotypeScore -A GenotypeSummaries -A GCContent -A FractionInformativeReads -A FisherStrand -A ExcessHet -A DepthPerSampleHC -A DepthPerAlleleBySample -A Coverage -A ClippingRankSumTest -A ChromosomeCounts -A BaseQualitySumPerAlleleBySample -A BaseQualityRankSumTest -A BaseCountsBySample -A BaseCounts -A AlleleCountBySample -A AlleleBalanceBySample

VarDict

VarDictJava/build/install/VarDict/bin/VarDict -b [individual].hs37d5.300x.bam.dedup.bam.RG.BQSR.bam.down0.1.bam -G hs37d5.fa -f 0.05 -Q 1 -q 15 -th 100 -P 1 -m 15 -c 1 -S 2 -E 3 [individual]_GRCh37_GIAB_highconf_CG-IllFB-IllGATKHC-Ion-10X-SOLID_CHROM1-22_v.3.3.2_[sample-specific high confident regions].bed | VarDictJava/VarDict/teststrandbias.R > individual.NHGRI_Illumina300X.down01_HC.3.3.2.vardict

**#rename sample in vcf file**

sed 's/.hs37d5.300x.bam.dedup.bam.RG.BQSR.bam.down0.1//g' [individual].NHGRI_Illumina300X.down01_HC.3.3.2.vardict > individual.NHGRI_Illumina300X.down01_HC.3.3.2.vardict.renamed

Edit header of ground truth vcf

Headers of ground truth vcf files need to be edited to work with hap.py.

zcat HG001_GRCh37_GIAB_highconf_CG-IllFB-IllGATKHC-Ion-10X-SOLID_CHROM1-X_v.3.3.2_highconf_PGandRTGphasetransfer.vcf.gz | perl -pe 's/^##FORMAT=<ID=PS,Number=1,Type=Integer,/##FORMAT=<ID=PS,Number=1,Type=String,/' | bcftools/bcftools view -o NA12878.GRCh37_GIAB_highconf_CG-IllFB-IllGATKHC-Ion-10X-SOLID_CHROM1-X_v.3.3.2_highconf.reheader.vcf.gz -O z

zcat HG002_GRCh37_GIAB_highconf_CG-IllFB-IllGATKHC-Ion-10X-SOLID_CHROM1-22_v.3.3.2_highconf_triophased.vcf.gz | perl -pe 's/^##FORMAT=<ID=PS,Number=1,Type=Integer,/##FORMAT=<ID=PS,Number=1,Type=String,/' | bcftools/bcftools view -o HG002.GRCh37_GIAB_highconf_CG-IllFB-IllGATKHC-Ion-10X-SOLID_CHROM1-22_v.3.3.2_highconf.reheader.vcf.gz -O z

**#rename sample in ground truth vcf - HG001 to NA12878**

gunzip NA12878.GRCh37_GIAB_highconf_CG-IllFB-IllGATKHC-Ion-10X-SOLID_CHROM1-X_v.3.3.2_highconf.reheader.vcf.gz

sed -i 's/HG001/NA12878/g' NA12878.GRCh37_GIAB_highconf_CG-IllFB-IllGATKHC-Ion-10X-SOLID_CHROM1-X_v.3.3.2_highconf.reheader.vcf

bgzip NA12878.GRCh37_GIAB_highconf_CG-IllFB-IllGATKHC-Ion-10X-SOLID_CHROM1-X_v.3.3.2_highconf.reheader.vcf

tabix NA12878.GRCh37_GIAB_highconf_CG-IllFB-IllGATKHC-Ion-10X-SOLID_CHROM1-X_v.3.3.2_highconf.reheader.vcf.gz

Create intersect of HC intervals between both individuals

The HC regions of both genomes were intersected using bedtools2 to create common HC regions shared by both individuals (NA12878_HG002.HC_intersect.bed), allowing a more precise estimation of pipeline performance. Quality metrics were calculated as an average between NA12878 and HG002 genomes. Variant counts represent their sum between both individuals.

bedtools2/bin/bedtools intersect -a HG001_GRCh37_GIAB_highconf_CG-IllFB-IllGATKHC-Ion-10X-SOLID_CHROM1-X_v.3.3.2_highconf_nosomaticdel.bed -b HG002_GRCh37_GIAB_highconf_CG-IllFB-IllGATKHC-Ion-10X-SOLID_CHROM1-22_v.3.3.2_highconf_noinconsistent.bed > NA12878_HG002.HC_intersect.bed

Variant filtering optimization

GATK HaplotypeCaller VQSR SNV step1

(Total combinations: 2)

**CLI call:** java -jar GenomeAnalysisTK.jar

**Input files extension:** raw.01.vcf

**Output files extension:** output

**Template:**

#!/bin/bash

FNAME=$(basename "[input_file_name]");

touch [output_files_dir]${FNAME}.[output_files_ext]

echo [cli_call] -T VariantRecalibrator [params] -input [input_file_name] -recalFile [output_files_dir]${FNAME}.[output_files_ext].recal -tranchesFile [output_files_dir]${FNAME}.[output_files_ext].tranches 2>[output_files_dir]${FNAME}.log2 --log_to_file [output_files_dir]${FNAME}.log -resource:dbsnp,known=true,training=false,truth=false,prior=2.0 dbsnp_138.b37.vcf -resource:1000G,known=false,training=true,truth=false,prior=10.0 1000G_phase1.snps.high_confidence.b37.vcf -resource:omni,known=false,training=true,truth=true,prior=12.0 1000G_omni2.5.b37.vcf -resource:hapmap,known=false,training=true,truth=true,prior=15.0 hapmap_3.3.b37.vcf -tranche 100.0 -tranche 99.9 -tranche 99.8 -tranche 99.7 -tranche 99.6 -tranche 99.5 -tranche 99.4 -tranche 99.3 -tranche 99.2 -tranche 99.1 -tranche 99.0 -tranche 98.0 -tranche 97.0 -tranche 96.0 -tranche 95.0 -tranche 94.0 -tranche 93.0 -tranche 92.0 -tranche 91.0 -tranche 90.0

**Parameters:**

-an value: [MQ]

-an value: [MQRankSum]

-an value: [QD]

-mode value: [SNP]

-an value: [DP]

-an value: [SOR]

-an value: [FS]

-an value: [ReadPosRankSum]

--maxGaussians values: [4 , 8]

-nt value: [100]

-R value: [hs37d5.fa]

GATK HaplotypeCaller VQSR SNV step2

(Total combinations: 20)

**CLI call:** java -jar GenomeAnalysisTK.jar

**Input files extension:** output

**Output files extension:** filter.vcf

**Template:**

#!/bin/bash

FNAME=$(basename "[input_file_name]");

NAME=$(echo $FNAME | cut -f1 -d '.');

echo [cli_call] -T ApplyRecalibration [params] -input ${NAME}*haplotyper.raw.01.vcf -recalFile [input_files_dir]${FNAME}.recal -tranchesFile [input_files_dir]${FNAME}.tranches -o [output_files_dir]${FNAME}.[output_files_ext] 2>[output_files_dir]${FNAME}.log2 --log_to_file [output_files_dir]${FNAME}.log

**Parameters:**

-mode SNP

--ts_filter_level values: [100.0 , 99.9 , 99.8 , 99.7 , 99.6 , 99.5 , 99.4 , 99.3 , 99.2 , 99.1 , 99.0 , 98.0 , 97.0 , 96.0 , 95.0 , 94.0 , 93.0 , 92.0 , 91.0 , 90.0]

-R value: [hs37d5.fa]

GATK HaplotypeCaller VQSR INDEL step1

(Total combinations: 2)

**CLI call:** java -jar GenomeAnalysisTK.jar

**Input files extension:** raw.01.vcf

**Output files extension:** output

**Template:**

#!/bin/bash

FNAME=$(basename "[input_file_name]");

touch [output_files_dir]${FNAME}.[output_files_ext]

echo [cli_call] -T VariantRecalibrator [params] -input [input_file_name] -recalFile [output_files_dir]${FNAME}.[output_files_ext].recal -tranchesFile [output_files_dir]${FNAME}.[output_files_ext].tranches 2>[output_files_dir]${FNAME}.log2 --log_to_file [output_files_dir]${FNAME}.log -tranche 100.0 -tranche 99.9 -tranche 99.8 -tranche 99.7 -tranche 99.6 -tranche 99.5 -tranche 99.4 -tranche 99.3 -tranche 99.2 -tranche 99.1 -tranche 99.0 -tranche 98.0 -tranche 97.0 -tranche 96.0 -tranche 95.0 -tranche 94.0 -tranche 93.0 -tranche 92.0 -tranche 91.0 -tranche 90.0 -resource:mills,known=false,training=true,truth=true,prior=12.0 Mills_and_1000G_gold_standard.indels.b37.vcf -resource:dbsnp,known=true,training=false,truth=false,prior=2.0 dbsnp_138.b37.vcf

**Parameters:**

-an value: [MQRankSum]

-an value: [QD]

--maxGaussians values: [4 , 8]

-nt value: [100]

-mode value: [INDEL]

-an value: [DP]

-an value: [SOR]

-an value: [FS]

-an value: [ReadPosRankSum]

-R value: [hs37d5.fa]

GATK HaplotypeCaller VQSR INDEL step2

(Total combinations: 20)

**CLI call:** java -jar GenomeAnalysisTK.jar

**Input files extension:** output

**Output files extension:** filter.vcf

**Template:**

#!/bin/bash

FNAME=$(basename "[input_file_name]");

NAME=$(echo $FNAME | cut -f1 -d '.');

echo [cli_call] -T ApplyRecalibration [params] -input ${NAME}*haplotyper.raw.01.vcf -recalFile [input_files_dir]${FNAME}.recal -tranchesFile [input_files_dir]${FNAME}.tranches -o [output_files_dir]${FNAME}.[output_files_ext] 2>[output_files_dir]${FNAME}.log2 --log_to_file [output_files_dir]${FNAME}.log

**Parameters:**

-mode [INDEL]

--ts_filter_level values: [100.0 , 99.9 , 99.8 , 99.7 , 99.6 , 99.5 , 99.4 , 99.3 , 99.2 , 99.1 , 99.0 , 98.0 , 97.0 , 96.0 , 95.0 , 94.0 , 93.0 , 92.0 , 91.0 , 90.0]

-R value: [hs37d5.fa]

VarDict

(Total combinations: 216)

**CLI call:** VarDictJava/VarDict/var2vcf_valid.pl

**Input files extension:** vardict.renamed

**Output files extension:** vcf

**Template:**

#!/bin/bash

FNAME=$(basename "[input_file_name]");

echo cat [input_file_name] | [cli_call] [params] > [output_files_dir]${FNAME}.[output_files_ext] 2>[output_files_dir]${FNAME}.log

**Parameters:**

-Q values: [default, 5 , 50]

-c values: [default , 3]

-p values: [default , 10 , 15]

-q values: [default, 30]

-f values: [default, 0.1 , 0.2]

-m values: [default , 6]

Post-processing

The filtered variants were compressed using bgzip and compared to the ground truth by RTG Tools in given ROI. Information about the pipelines’ performance (precision, recall, F-measure, etc.) was stratified into variant sub-types and genomic regions by hap.py. The results were imported into the ToTem’s internal database and filtered using ToTem’s filtering tool, allowing the selection of the best performing pipeline based on region, variant type and quality metrics.

Bgzip - vcf compression

(Total combinations: 1)

**CLI call:** bgzip

**Input files extension:** vcf

**Output files extension:** gz

**Template:**

#!/bin/bash

FNAME=$(basename "[input_file_name]");

[cli_call] [params] [input_file_name] > [output_files_dir]${FNAME}.[output_files_ext] 2>[output_files_dir]${FNAME}.log

tabix [output_files_dir]${FNAME}.[output_files_ext]

**Parameters:**

-c

RTG Tools - comparison of filtered variants to the ground truth

(Total combinations: 1)

**CLI call:** java -jar rtg-tools-3.7-23b7d60/RTG.jar vcfeval

**Input files extension:** gz

**Output files extension:** dir

**Template:**

#!/bin/bash

FNAME=$(basename "[input_file_name]");

NAME=$(echo $FNAME | cut -f1 -d '.');

echo [cli_call] [params] -c [input_file_name] -o [output_files_dir]${FNAME}.[output_files_ext] --sample $NAME --bed-regions NA12878_HG002.HC_intersect.bed -b $NAME*highconf.reheader.vcf.gz

**Parameters:**

--ref-overlap

-m value: [ga4gh]

--threads value: [50]

-t value: [hs37d5.SDF/]

hap.py - variant type and region stratification

(Total combinations: 1)

**CLI call:** python hap.py/hap.py-build/bin/qfy.py

**Input files extension:** dir

**Output files extension:** extended.csv

**Template:**

#!/bin/bash

FNAME=$(basename "[input_file_name]");

NAME=$(echo $FNAME | cut -f1 -d '.');

echo [cli_call] [params] -o [output_files_dir]${FNAME}.qfy --logfile [output_files_dir]${FNAME}.log [input_file_name]/output.vcf.gz -f NA12878_HG002.HC_intersect.bed

**Parameters:**

--stratification value: [stratification-bed-files_20160913/files.tsv

-V

-t value: [ga4gh]

--verbose

-X

-r value: [hs37d5.fa]

**#Default settings were tested only for variant filtration (both variant callers), because GATK HaplotypeCaller has not generated results using default parameters for raw variant calling in case of NA12878. Default VQSR setup correspond to GATK “Best practices”.**

# References

1. Malcikova J, Stano-Kozubik K, Tichy B, Kantorova B, Pavlova S, Tom N, et al. Detailed analysis of therapy-driven clonal evolution of TP53 mutations in chronic lymphocytic leukemia. Leukemia. 2015;29:877–85.

2. Gerstung M, Papaemmanuil E, Campbell PJ. Subclonal variant calling with multiple samples and prior knowledge. Bioinformatics. 2014;30:1198–204.

3. Kubesova B, Pavlova S, Malcikova J, Kabathova J, Radova L, Tom N, et al. Low-burden TP53 mutations in chronic phase of myeloproliferative neoplasms: association with age, hydroxyurea administration, disease type and JAK2 mutational status. Leukemia. 2017. http://dx.doi.org/10.1038/leu.2017.230.

4. Zook JM, Catoe D, McDaniel J, Vang L, Spies N, Sidow A, et al. Extensive sequencing of seven human genomes to characterize benchmark reference materials. Scientific Data. 2016;3:sdata201625.

5. Quinlan AR, Hall IM. BEDTools: a flexible suite of utilities for comparing genomic features. Bioinformatics. 2010;26:841–2.

6. Li H, Durbin R. Fast and accurate short read alignment with Burrows–Wheeler transform. Bioinformatics. 2009;25:1754–60.

7. Li H, Handsaker B, Wysoker A, Fennell T, Ruan J, Homer N, et al. The Sequence Alignment/Map format and SAMtools. Bioinformatics. 2009;25:2078–9.

8. DePristo MA, Banks E, Poplin RE, Garimella KV, Maguire JR, Hartl C, et al. A framework for variation discovery and genotyping using next-generation DNA sequencing data. Nat Genet. 2011;43:491–8.

9. Van der Auwera GA, Carneiro MO, Hartl C, Poplin R, del Angel G, Levy-Moonshine A, et al. From FastQ data to high confidence variant calls: the Genome Analysis Toolkit best practices pipeline. Curr Protoc Bioinformatics. 2013;11:11.10.1-11.10.33.

10. hap.py: Haplotype VCF comparison tools. C++. Illumina; 2017. https://github.com/Illumina/hap.py. Accessed 18 Dec 2017.

11. picard: A set of command line tools (in Java) for manipulating high-throughput sequencing (HTS) data and formats such as SAM/BAM/CRAM and VCF. Java. Broad Institute; 2017. https://github.com/broadinstitute/picard. Accessed 22 Dec 2017.

12. rtg-tools: RTG Tools: Utilities for accurate VCF comparison and manipulation. Java. Real Time Genomics; 2017. https://github.com/RealTimeGenomics/rtg-tools. Accessed 18 Dec 2017.

13. Lai Z, Markovets A, Ahdesmaki M, Chapman B, Hofmann O, McEwen R, et al. VarDict: a novel and versatile variant caller for next-generation sequencing in cancer research. Nucleic Acids Res. 2016;44:e108.

14. Koboldt DC, Chen K, Wylie T, Larson DE, McLellan MD, Mardis ER, et al. VarScan: variant detection in massively parallel sequencing of individual and pooled samples. Bioinformatics. 2009;25:2283–5.

15. Koboldt DC, Zhang Q, Larson DE, Shen D, McLellan MD, Lin L, et al. VarScan 2: somatic mutation and copy number alteration discovery in cancer by exome sequencing. Genome Res. 2012;22:568–76.

16. vcflib: a simple C++ library for parsing and manipulating VCF files, + many command-line utilities. C++. vcflib; 2017. https://github.com/vcflib/vcflib. Accessed 22 Dec 2017.
